# YTHDF2 reduction fuels inflammation and vascular abnormalization in hepatocellular carcinoma

**DOI:** 10.1186/s12943-019-1082-3

**Published:** 2019-11-18

**Authors:** Jiajie Hou, He Zhang, Jun Liu, Zhenjun Zhao, Jianye Wang, Zhike Lu, Bian Hu, Jiankui Zhou, Zhicong Zhao, Mingxuan Feng, Haiyan Zhang, Bin Shen, Xingxu Huang, Beicheng Sun, Chuan He, Qiang Xia

**Affiliations:** 1 Department of Liver Surgery, Renji Hospital, School of Medicine, Shanghai Jiaotong University, Shanghai, 200127 China; 2 Department of Hepatobiliary Surgery, The Affiliated Drum Tower Hospital of Nanjing University Medical School, Nanjing, 210093 China; 3 Department of Hepatobiliary Surgery, Sun Yat-sen University Cancer Center, Guangzhou, 510060 China; 4 State Key Laboratory of Oncology in South China, Sun Yat-sen University Cancer Center, Guangzhou, 510060 China; 5 Department of Surgery, The University of Hong Kong-Shenzhen Hospital, Shenzhen, 518053 China; 6 Department of Chemistry, Department of Biochemistry and Molecular Biology, Institute for Biophysical Dynamics, University of Chicago, Chicago, IL 60637 USA; 7 School of Life Science and Technology, ShanghaiTech University, Shanghai, 201210 China; 8 Immunology of Cancer and Infection Laboratory, QIMR Berghofer Medical Research Institute, Herston, Queensland 4006 Australia; 9 Key Laboratory of Reproductive Medicine, Department of Histology and Embryology, Nanjing Medical University, Nanjing, 211166 China; 10 Howard Hughes Medical Institute, University of Chicago, Chicago, IL 60637 USA

**Keywords:** m^6^A, YTHDF2, HCC, Inflammation, Vessel normalization, IL-11, Serpin E2, HIF-2α antagonism

## Abstract

**Background:**

Dynamic *N*^6^-methyladenosine (m^6^A) modification was previously identified as a ubiquitous post-transcriptional regulation that affected mRNA homeostasis. However, the m^6^A-related epitranscriptomic alterations and functions remain elusive in human cancer. Here we aim to identify the profile and outcome of m^6^A-methylation in hepatocellular carcinoma (HCC).

**Results:**

Using liquid chromatography-tandem mass spectrometry and m^6^A-immunoprecipitation in combination with high-throughput sequencing, we determined the m^6^A-mRNA levels in human HCC. Human HCC exhibited a characteristic gain of m^6^A modification in tandem with an increase of mRNA expression, owing to YTH domain family 2 (YTHDF2) reduction. The latter predicted poor classification and prognosis of HCC patients, and highly correlated with HCC m^6^A landscape. YTHDF2 silenced in human HCC cells or ablated in mouse hepatocytes provoked inflammation, vascular reconstruction and metastatic progression. Mechanistically, YTHDF2 processed the decay of m^6^A-containing interleukin 11 (IL11) and serpin family E member 2 (SERPINE2) mRNAs, which were responsible for the inflammation-mediated malignancy and disruption of vascular normalization. Reciprocally, YTHDF2 transcription succumbed to hypoxia-inducible factor-2α (HIF-2α). Administration of a HIF-2α antagonist (PT2385) restored YTHDF2-programed epigenetic machinery and repressed liver cancer.

**Conclusion:**

Our results have characterized the m^6^A-mRNA landscape in human HCC and revealed YTHDF2 as a molecular ‘rheostat’ in epitranscriptome and cancer progression.

## Background

As the most frequent internal decoration on eukaryotic mRNAs, *N*^6^-methyladenosine (m^6^A) has been proven to play fundamental roles in regulating gene expression and biological processes [1–3]. Pioneering studies have offered a glimpse into the sophisticated mechanisms of reversible m^6^A modification in cancer. The demethylase FTO (fat mass-and obesity-associated protein) decreases global m^6^A levels, leading to either downregulation of tumor suppressor genes or upregulation of tumor promoter genes [4, 5]. Another demethylase ALKBH5 (a-ketoglutarate-dependent dioxygenase alkB homolog 5) tends to stabilize stem-cell-related transcripts [6, 7]. METTL3 (methyltransferase-like 3)-catalyzed RNA methylation is required for cancer development [8–12], whereas the role of METTL14 varies with cancer types [13–16]. Diverse post-transcriptional outcomes of m^6^A-methylation rely on its readers, which are poorly understood in human cancers. YTHDF2 (YTH domain family 2) is the most effective m^6^A reader that weakens mRNA stability by recognizing and distributing m^6^A-containing mRNAs to processing bodies [17, 18]. Recent reports referred YTHDF2 was able to degrade both tumor promoter and suppressor gene mRNAs [5, 8, 16, 19, 20]. While it enhanced self-renewing of leukemic stem cells [20], the basic role of endogenous YTHDF2 in solid cancer remained in question.

Despite the identification of thousands of m^6^A sites in a number of human cancer cell lines, less is known about the epigenomic alterations during cancer progression and the epitranscriptomic features amongst patients with any particular type of cancer. The metabolic traits of cancer cells, which are critical for adapting to the changes within the tumor microenvironment, often contribute to the epigenetic plasticity. As one of the most well-recognized cancer hallmarks, hypoxia can affect DNA methylation and histone methylation or acetylation by reducing enzymatic activity, regulating gene expression or increasing metabolite production [21, 22]. In terms of m^6^A-mRNA editing, hypoxia-inducible factors (HIFs) upregulate ALKBH5 expression, which in turn leads to NANOG mRNA demethylation in breast cancer cells [7]. Nonetheless, the oncometabolite 2-hydroxyglutarate (2HG) attenuates FTO activity, thereby increasing global m^6^A RNA modification in 2HG-sensitive leukemia or glioma cells [5, 23]. Another study reported that hypoxia broadly increases m^6^A modification to mRNAs, leading to the acquisition of higher mRNA stability instead of m^6^A-mediated decay [24]. These findings all suggest that tumor hypoxia causes m^6^A epigenetic remodeling, but the prevalent alterations and the molecule which triggers these alterations have not yet been determined.

Hepatocellular carcinoma (HCC), which possesses the hallmarks of hypoxia and chronic inflammation, is one of the most lethal malignancies worldwide [25, 26]. Here we identify a characteristic gain of m^6^A modification in tandem with an increase of mRNA expression in both human HCC specimens and cell lines exposed to hypoxia. In an epitranscriptome-wide setting, these “hyper-upregulated” genes exhibit a remarkable association with molecular rewiring for cancer progression. The reduction of YTHDF2, which predicts poor classification and prognosis of HCC patients, highly correlates with this epitranscriptional orchestration and promotes tumor growth and metastasis. Through genetic remodeling and m^6^A-mRNA profiling, we link YTHDF2 deficiency to mRNA stabilization of IL-11 and Serpin E2, the key mediators in support of hypoxia-induced cancer cell survival and vascular reconstruction [27, 28]. We further reveal a transcriptional inhibition of YTHDF2 by HIF-2α, which has been therapeutically targeted in renal cell carcinoma and HCC [29, 30]. Administration of a first-in-class HIF-2α antagonist (PT2385) [31] restored and required YTHDF2 to suppress inflammation-associated cancer progression. Together, our data highlight a protective role of YTHDF2 in HCC interrupting the hypoxia-induced epigenetic-inflammation-cancer axis.

## Results

### Human HCC manifests a ‘hyper-up’ alteration in m^6^A-mRNA landscape

**Fig. 1:**
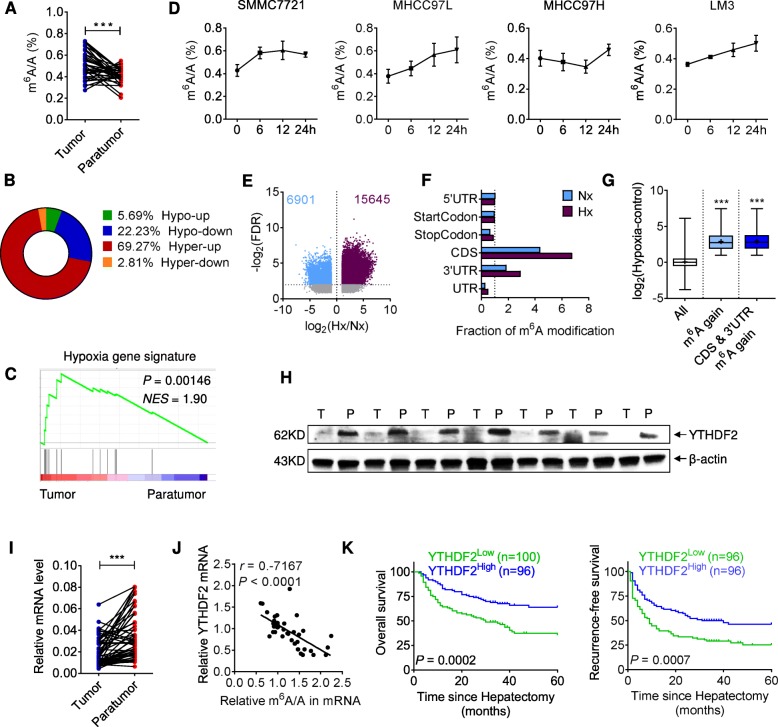
Hyper-upregulation of m^6^A-mRNAs and downregulation of YTHDF2 in human HCC. (**a**) m^6^A levels in paired tumor and paratumor mRNA, as assessed using LC-MS/MS. *n* = 37 patients. (**b**) Percentages of altered m^6^A-mRNAs (classified as hypo-up, hypo-down, hyper-up and hyper-down) in tumor compared to paratumor, as determined by MeRIP-Seq. *n* = 8 patients. (**c**) Gene-set enrichment analysis of hypoxia-signature gene expression in paired tumor versus paratumor mRNA, as determined by RNA-seq. *n* = 8 patients. NES, normalized enrichment score. (**d**) m^6^A levels in mRNAs of human HCC cell lines grown for 0, 6, 12 or 24 h under hypoxia, as assessed using LC-MS/MS. *n* = 2 independent experiments. (**e**) Changes in m^6^A peaks of SMMC7721 cells upon 24 h of hypoxia (Hx) versus normoxia (Nx), as detected using MeRIP-Seq. Peaks gaining (violet) or losing (light blue) m^6^A are highlighted at *P* < 0.05 and 5% FDR adjustment. (**f**) Fraction of m^6^A peaks in diverse transcript segments, under either hypoxic or normoxic condition. (**g**) Expression changes of mRNAs in hypermethylated peaks. (**h**) Immunoblot of YTHDF2 in paired tumor (T) and paratumor (P) tissues. *n* = 7 patients. (**i**) YTHDF2 mRNA levels in paired tumor and paratumor tissues, as assessed by RT-qPCR. *n* = 51 patients. (**j**) Scatter plots showing the correlation between m^6^A level and YTHDF2 expression. The linear best fit line, Pearson correlation coefficient (*r*) and *P*-value (*P*) are shown. *n* = 37 patients. (**k**) Kaplan-Meier analyses of the correlation between YTHDF2 expression level and overall survival (left) or recurrence-free survival (right) in HCC patients. *n* = 200 patients in total. Error bars indicate means ± SEM *P < 0.05, **P < 0.01, ***P < 0.001, **** *P* < 0.0001. *P*-values were determined by two tailed *t*-test

Despite the m^6^A decrease in total RNA of human HCC, there is still no consensus about mRNA modification [8, 13]. To assess the global m^6^A-methylation in HCC, we examined 37 tumor paired with adjacent non-tumor samples. By performing dot blot or liquid chromatography-tandem mass spectrometry (LC-MS/MS), we observed a slight m^6^A decrease in total RNA, and yet a significant m^6^A increase in mRNA, while comparing tumor tissues with autologous paratumor tissues (Additional file 1: Figure S1A, B and Fig. 1a). Next, eight pairs of mRNA samples were immunoprecipitated using an m^6^A-specific antibody in combination with high-throughput sequencing (MeRIP-seq). An average of 70.80 and 29.20% m^6^A peaks showed a significant increase and decrease, respectively, in tumor relative to non-tumor samples (Additional file 1: Figure S1C). Through analysis of RNA sequencing (RNA-seq) data, we noticed most transcripts that gained in m^6^A manifested higher mRNA expression in tumor versus non-tumor counterparts. The superiority of ‘hyper-up’ population was found in 7 out of 8 patients, averagely taking up 69.27% amongst all the m^6^A-labelled transcripts (Fig. 1b and Additional file 1: Figure S1D, E). We then analyzed those high-frequency ‘hyper-up’ genes (at least shared by 3 patients), which are mainly responsible for cancer-promoting events, as indicated by Gene ontology (GO) analysis (Additional file 1: Figure S1F).

Using gene set enrichment analysis (GSEA), a previously established hypoxia signature [32] was strongly skewed toward tumor counterparts in the case of HCC (Fig. 1c). A recent study suggested hypoxia stimulated m^6^A demethylation^6^ whereas another report showed hypoxia favored m^6^A labelling [24]. Therefore, we exposed HCC cell lines to hypoxia and observed a gain of m^6^A in all four cell lines as compared to normoxic conditions (Fig. 1d). Using MeRIP-seq, we detected a larger number of m^6^A peaks in hypoxic SMMC7721 mRNA, preferentially enriched in 3′ untranslated regions (3’UTR) and coding regions (CDS) (Fig. 1e, f). As profiled by RNA-seq, transcripts gained in m^6^A upon hypoxia manifested profoundly increased expression (Fig. 1g), which recapitulated the ‘hyper-up’ pattern identified in HCC specimens. GO analysis of those ‘hyper-up’ genes suggested activation of hypoxia-related oncogenic pathways (Additional file 1: Figure S1G). Notably, gene signatures of cell-cell contact and ‘serine-type endopeptidase inhibitor activity’ are enriched in ‘hyper-up’ populations based on both tumor tissue and hypoxic cell profiling (Additional file 1: Figure S1F, G).

### YTHDF2 is associated with HCC epitranscriptome and clinical outcome

To analyze how hypoxia was linked to m^6^A-mRNA editing, we measured enzyme expression in HCC tissues as well as hypoxic cancer cells. Among methyltransferases, METTL14 mRNA displayed significant alteration in tumor (Additional file 1: Figure S2A), and yet its decrease could not explain the observed increase in global m^6^A. In concert with m^6^A hypermethylation, FTO and ALKBH5 mRNAs decreased in tumor tissues; however, they both increased under hypoxic conditions (Additional file 1: Figure S2A, B). Notably, YTHDF2 mRNA was reduced in both hypoxic cells and tumor tissues, which indicated an inverse correlation with the m6A/A ratio (Additional file 1: Figure S1H and 1I, J). Putatively, ‘hyper-up’ regulation of the m^6^A-epitranscriptome in HCC might result from the YTHDF2 reduction and consequential m^6^A-mRNA stabilization. We verified YTHDF2 protein levels were also decreased in hypoxic cells and tumor specimens (Additional file 1: Figure S1I, 1H and S2C). Importantly, in a setting of 200 HCC patients (recruited from 2006 to 2012), lower YTHDF2 protein levels were significantly associated with more multinodular tumors and microvascular invasion, higher TNM and BCLC stage classification, and shorter overall and recurrence-free survival period (Additional file 2: Table S1, Additional file 1: Figure S2D and 1 K). Accordingly, we hypothesized that a hypoxia-sensitive YTHDF2 reduction reprogrammed the m^6^A-edited transcriptome and promoted HCC development.

### YTHDF2 deficiency promotes HCC growth, vasculature remodeling and metastasis

**Fig. 2:**
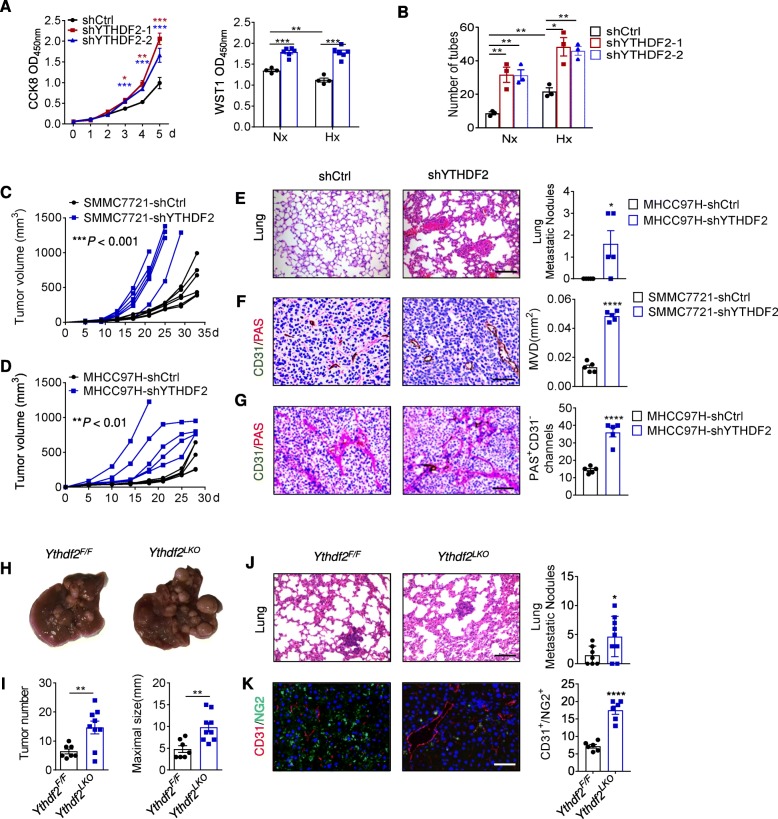
YTHDF2 deficiency promotes tumor growth, vasculature remodeling and metastasis. (**a**) Proliferative activity of SMMC7721-shCtrl and SMMC7721-shYTHDF2 cells grown for 5 d as assessed by CCK8 (left), or grown for additional 24 h of normoxia (Nx) or hypoxia (Hx) and assessed by WST1 assay (right). *n* = 4–6 biological replicates. (**b**) Numbers of endothelial tubes formed by HUVEC cocultured with indicated SMMC7721 cells under Nx or Hx for 24 h. *n* = 3 biological replicates. (**c**, **d**) Indicated SMMC7721 (**c**) or MHCC97H (**d**) cells were injected subcutaneously into NPG mice and tumor volumes were measured at indicated dates. *n* = 5–6 mice. (**e**) H&E staining of lung tissues, showing metastasis nodules originated from MHCC97H cells. *n* = 5 mice. Scale bar, 100 μm. (**f**) Microvessel density (MVD) in SMMC7721-derived tumors, as assessed by CD31 staining and quantification of microvessel areas. *n* = 5 mice. Scale bar, 100 μm. (**g**) Vascular mimicry in MHCC97H-derived tumors, as quantified by numbers of PAS^+^CD31^−^ channels lined by tumor cells. *n* = 5 mice. Scale bar, 100 μm. (**h**-**j**) Macropscopic appearances (**h**), tumor numbers (**i**) and maximum tumor sizes of livers from 8.5-month old *Ythdf2*^*F/F*^ and *Ythdf2*^*LKO*^ mice injected with DEN and CCL4. *n* = 7–9 mice. (**j**) H&E staining of metastasis nodules in *Ythdf2*^*F/F*^ and *Ythdf2*^*LKO*^ mouse lungs. *n* = 7–9 mice. Scale bar, 50 μm. (**k**) Immunofluorescence staining of CD31 and NG2, showing microvessels and pericyte coverage in *Ythdf2*^*F/F*^ and *Ythdf2*^*LKO*^ mouse livers. *n* = 7–9 mice. Scale bar, 50 μm. Error bars indicate means ± SEM *P < 0.05, **P < 0.01, ***P < 0.001, **** *P* < 0.0001. *P*-values were determined by two tailed *t*-test

A recent report has preliminarily showed that overexpression of YTHDF2 could suppress HCC growth [19], but the importance of endogenous YTHDF2 in HCC cells was still not clear. Hence, we stably silenced YTHDF2 expression in human HCC cell line SMMC7721 and MHCC97H (Additional file 1: Figure S3A, B), and assessed their biological behaviors. Disruption of YTHDF2 greatly benefited cell viability but did not retard apoptosis (Fig. 2a and Additional file 1: Figure S3C, D). Impressively, this acquired growth competence was conserved under hypoxic conditions (Fig. 2a). In terms of hypoxia-related events, we further evaluated cell migration, stemness, metabolism, and tumor angiogenesis [33]. YTHDF2 knockdown resulted in a promotion of angiogenic sprouting of human umbilical vein endothelial cells (HUVECs) in a co-culture system (Fig. 2b and Additional file 1: Figure S3E), but unaffected other cellular behaviors (Additional file 1: Fig. S3F-H). We next exploited a subcutaneous xenograft mouse model to monitor in vivo tumor development. YTHDF2 silencing robustly facilitated tumor growth and metastasis in NPG (NOD-*Prkdc*^scid^*Il2rg*^null^) mice (Fig. 2c-e), and this was partially due to the effects on cell proliferation and tumor angiogenesis (Additional file 1: Figure S3I, K and Fig. 2f). It is well-documented that tumor vascular permeability and mimicry contributes to extravasation and metastasis [28, 34]. In line with this, YTHDF2 silencing in MHCC97H tumors led to increased dextran leakage and PAS^+^CD31^−^ fluid-filled channels lined by tumor cells, indicating intensified vascular permeability and mimicry (Additional file 1: Figure S3J, L and Fig. 2g). In contrast, overexpression of YTHDF2 not only arrested cancer cell growth but also reduced vessel density and permeability (Additional file 1: Fig. S4A-H).

To obtain a deeper insight into the impact of YTHDF2 deficiency on HCC, we generated the liver-specific *Ythdf2* KO mouse strain (*Ythdf2*^LKO^) and employed a chemical-induced HCC model. As compared with *Ythdf2*^F/F^ littermates*, Ythdf2*^LKO^ mice developed more advanced liver lesions, in terms of macroscopic number, size and histology, and more lung metastases (Fig. 2h-j and Additional file 1: Figure S4I). Tumor proliferation, apoptosis and angiogenesis were enhanced in the YTHDF2-deficient mouse liver (Additional file 1: Figure S4J, K and Fig. 2k). Nonetheless, no obvious vascular mimicry existed in either *Ythdf2*^F/F^ or *Ythdf2*^LKO^ liver tumors (data not shown). To our knowledge, the microvascular network in solid tumors is often compromised with functional abnormalities, which fuel metastasis formation [35]. In the absence of YTHDF2, NG2^+^ pericytes were significantly reduced, whereas CD31^+^ endothelial cells largely accumulated and formed microvessels in tumor regions (Fig. 2k), defining a fundamental role for YTHDF2 in vascular normalization [35].

### YTHDF2 deficiency escalates inflammation and vessel abnormalization

**Fig. 3:**
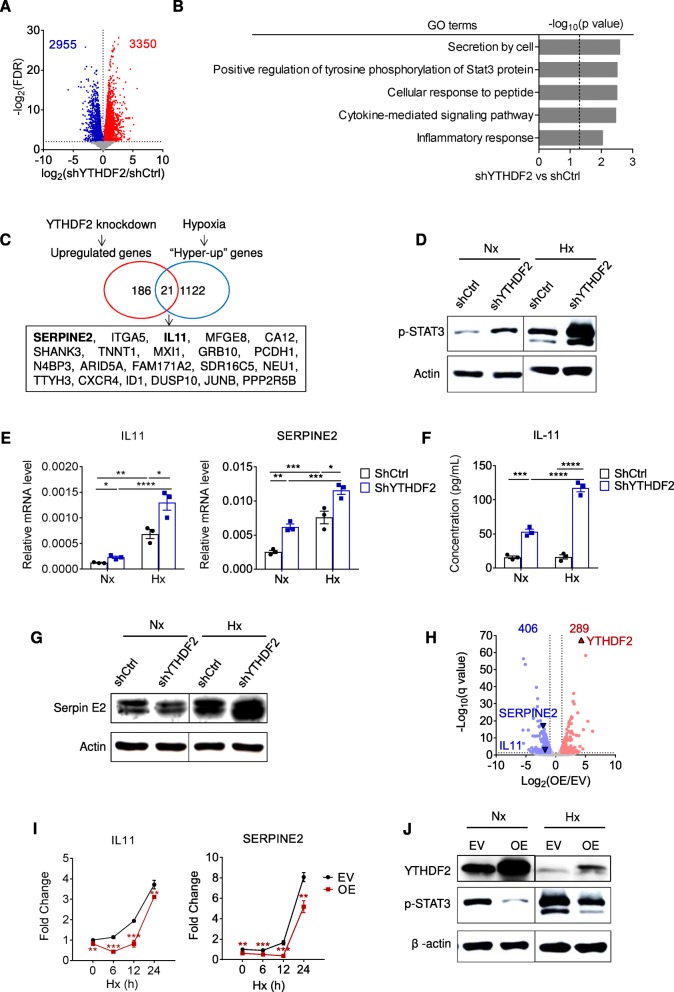
YTHDF2 inhibits IL-11 and Serpin E2 expression in HCC cells. (**a**) Volcano plots showing up (red) - or down (blue) -regulated genes in SMMC7721-shYTHDF2 versus SMMC7721-shCtrl cells, as assessed by RNA-seq. (**b**) GO analysis based on the RNA-seq in (A), showing the most significant GO terms and the *P* values. (**c**) Screening strategy showing a group of overlapped genes that were concomitant in YTHDF2-deficiency-induced upregulation and hypoxia-induced hyper-upregulation. (**d**, **g**) Immunoblots showing STAT3 phosphorylation (**d**) and Serpin E2 (**g**) expression in indicated SMMC7721 cells grown for 24 h under Nx or Hx. *n* = 2 independent experiments. (**e**) RT-qPCR analysis of the relative mRNA levels of IL11 (left) and SERPINE2 (right) in indicated SMMC7721 cells grown for 24 h under Nx or Hx. *n* = 3 biological replicates. (**f**) ELISA analysis of the quantitative protein levels of IL-11 in the culture supernatant of indicated SMMC7721 cells grown for 24 h under Nx or Hx. *n* = 3 biological replicates. (**h**) Volcano plots showing up (red) - or down (blue) -regulated genes in SMMC7721-OE versus SMMC7721-EV cells grown for 12 h under hypoxia, as assessed by RNA-seq. (**i**) RT-qPCR analysis of the relative mRNA levels of IL11 (left) and SERPINE2 (right) in SMMC7721-EV and SMMC7721-OE cells grown for 0, 6, 12 or 24 h under Hx. *n* = 3 biological replicates. (**j**) Immunoblots showing STAT3 phosphorylation in indicated SMMC7721 cells grown for 24 h under Nx or Hx. *n* = 2 independent experiments. Error bars indicate means ± SEM *P < 0.05, **P < 0.01, ***P < 0.001, **** *P* < 0.0001. *P*-values were determined by two tailed *t*-test

We subsequently performed RNA-seq in control and YTHDF2-deficient SMMC-7721 cells. Taking into consideration that putative targets were retarded for degradation, we mainly focused on the upregulated transcripts (Fig. 3a). GO analysis revealed an enrichment of a cancer-promoting inflammation program, as characterized by the top GO term ‘secretion by cell’ and ‘positive regulation of tyrosine phosphorylation of Stat3 protein’ (Fig. 3b and Additional file 3: Table S2). We did not see epidermal growth factor receptor (EGFR) among the upregulated genes, although it has been recently reported elsewhere [19]. To find out the key determinants in a hypoxic cancer context, we used a combined screening approach by integrating YTHDF2-regulated transcriptional profiling and the hypoxia-responsive epitranscriptome. Therefore, we selected 182 genes that were increased by over 2-fold upon YTHDF2 knockdown, and 1122 hypermethylated transcripts that were upregulated by over 2-fold under hypoxic conditions (Fig. 3c). Precisely, these genes intersected for 21 candidate targets, among which IL11 and SERPINE2 were most relevant to cancer-promoting inflammation. IL-11 promotes STAT3 activation and inflammatory cancer progression in an autocrine manner [27, 36], while cancer cell-secreted serpin E2 confers invasive and metastatic processes by reprogramming the tumor vasculature [28, 37–39]. We validated that both STAT3 phosphorylation and IL11 and SERPINE2 expression were upregulated in YTHDF2-deficient cells, and this effect could be reinforced by oxygen deficit (Fig. 3d-g and Additional file 1: Figure S5A-D). In parallel, YTHDF2 deficiency in mouse hepatocytes yielded IL11 and SERPINE2 expression (Additional file 1: Fig. S5E). Conversely, RNA profiling and qPCR detection of YTHDF2-overexpressed cells showed substantial downregulation of IL11 and SERPINE2, as well as phosphorylation of STAT3, in comparison to the control cells (Fig. 3i-j and Additional file 4: Table S3). Unexpectedly, short-term (12 h) exposure to hypoxia unleashed the repressive properties of YTHDF2 toward IL11 and SERPINE2 mRNAs (Fig. 3I).

### YTHDF2 degrades m^6^A-containing IL11 and SERPINE2 mRNAs

**Fig. 4:**
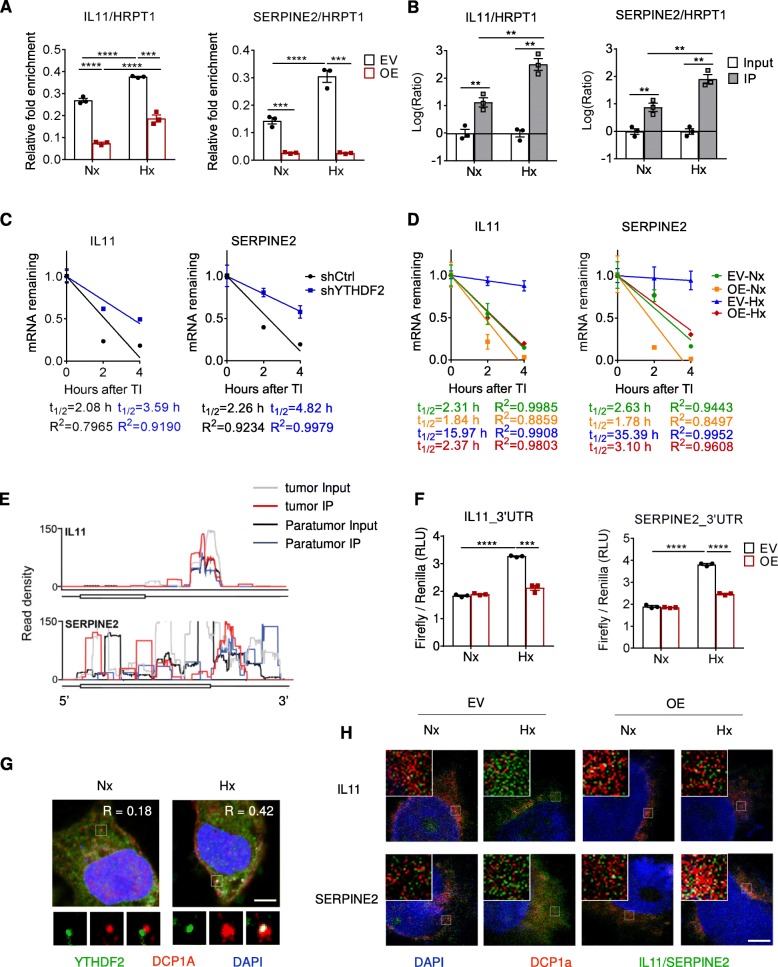
YTHDF2 processes m^6^A-marked IL11 and SERPINE2 mRNAs for decay. (**a**) m^6^A-enrichment in the IL11 and SERPINE2 mRNAs in SMMC7721 cells expressing empty vector (EV) or overexpressing YTHDF2 (OE), as assessed by MeRIP-qPCR. The cells were subjected to Nx or Hx for 12 h. *n* = 3 biological replicates. (**b**) Gene enrichment of IL11 and SERPINE2 in YTHDF2-mRNA complex immunoprecipitated from SMMC7721-OE cells, as determined by RIP-qPCR. The cells were subjected to Nx or Hx for 12 h. *n* = 3 biological replicates. (**c**, **d**) RNA lifetime of IL11 and SERPINE2 in indicated SMMC7721 cells, as determined by monitoring transcript abundance after transcription inhibition (TI). The cells were subjected to Nx or Hx 8 h prior to TI initiation. *n* = 3 biological replicates. (**e**) The average read density showing the m^6^A peaks identified in IL11 and SERPINE2 transcripts in human HCC tissues as assessed using MeRIP-seq. *n* = 8 patients. (**f**) Luciferase reporter assay showing posttranscriptional regulation by YTHDF2 in the presence of IL11 (left) or SERPINE2 (right) 3’UTR. Indicated SMMC7721 cells were grown for 12 h under Nx or Hx. *Renilla* luciferase activity was normalized to firefly activity and presented as relative luciferase activity. *n* = 3 biological replicates. (**g**) Immunofluorescence staining of YTHDF2 and DCP1a (P-body marker) in YTHDF2-overexpressing SMMC7721 cells grown for 12 h under Nx or Hx. *n* = 3 biological replicates. Scale bar, 5 μm. (**h**) Fluorescence in situ hybridization of IL11 or SERPINE2 mRNA and immunofluorescence staining of DCP1a in indicated SMMC7721 cells grown for 12 h under Nx or Hx. *n* = 3 biological replicates. Scale bar, 5 μm. Error bars indicate means ± SEM *P < 0.05, **P < 0.01, ***P < 0.001, **** *P* < 0.0001. *P*-values were determined by two tailed *t*-test

Using m^6^A-specific qPCR, we uncovered that YTHDF2 induced m^6^A loss in IL11 and SERPINE2 transcripts (Fig. 4a). To investigate whether YTHDF2 directly recognized m^6^A-marked IL11 and SERPINE2 mRNAs, we performed mRNA-immunoprecipitation (RIP) for YTHDF2. Subsequent RT-qPCR identified IL11 and SERPINE2 as YTHDF2 substrates, particularly under hypoxic condition (Fig. 4b). We then asked whether YTHDF2 processes IL11 and SERPINE2 mRNAs to decay. After actinomycin D treatment, their lifetimes were prolonged in YTHDF2-silenced cells and were shortened in YTHDF2-overexpressed cells (Fig. 4c, d). Although hypoxia dramatically delayed IL11 and SERPINE2 degradation in control cells, YTHDF2 overexpression expedited the processing to a normal level. Since human HCC tissues exhibited high abundance of m^6^A modification to the consensus sites of IL11 and SERPINE2 3’UTRs (Fig. 4e), we inserted their transcript segments harboring m^6^A motifs into a pmirGLO reporter plasmid [17]. Under hypoxic conditions, introduction of exogenous YTHDF2 efficiently disrupted reporter activity in the presence of m^6^A motifs (Fig. 4f). Using fluorescence in situ hybridization (FISH), we visualized localization of YTHDF2 and target mRNAs to processing bodies (Fig. 4g, h). As expected, YTHDF2 affected both expression and distribution of IL11 and SERPINE2 mRNAs. Noticeably, 12 h of exposure to hypoxia induced YTHDF2 translocation to processing bodies, substantially supporting its provisional potency propelled by a lack of oxygen (Fig. 4g).

**Fig. 5:**
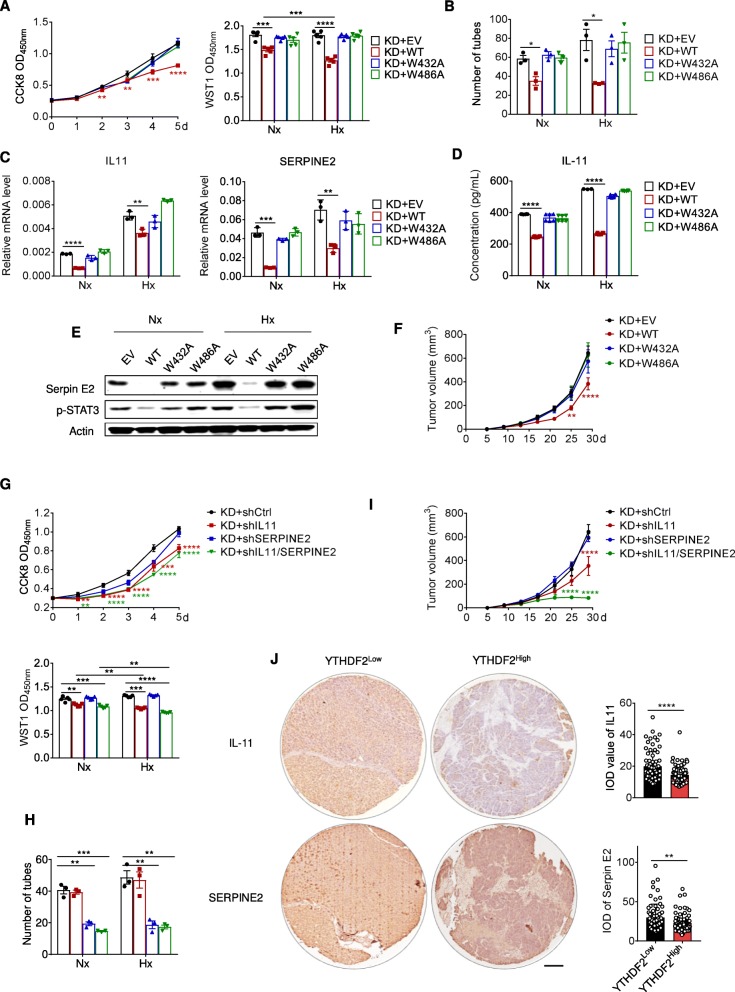
YTHDF2 suppresses HCC by targeting IL11 and SERPINE2 mRNAs in a m^6^A-reader fashion. (**a**) An empty vector (EV) or vectors encoding wild-type (WT) or mutant YTHDF2 (W432A and W486A) were transduced into SMMC7721 cells with a YTHDF2-knockdown (KD) background. Proliferative activity of indicated SMMC7721 cells was assessed by CCK8 (left) or WST1 assay (right). *n* = 5 biological replicates. (**b**) Numbers of endothelial tubes formed by HUVEC cocultured with indicated SMMC7721 cells grown for 24 h under Nx or Hx. *n* = 3 biological replicates. (**c**) RT-qPCR analysis of the relative mRNA levels of IL11 (left) and SERPINE2 (right) in indicated SMMC7721 cells grown for 24 h under Nx or Hx. *n* = 3 biological replicates. (**d**) ELISA analysis of the quantitative protein levels of IL-11 in the culture supernatant in indicated SMMC7721 cells grown for 24 h under Nx or Hx. *n* = 3 biological replicates. (**e**) Immunoblots showing STAT3 phosphorylation and Serpin E2 expression in indicated SMMC7721 cells grown for 24 h under Nx or Hx. *n* = 2 independent experiments. (**f**) Indicated SMMC7721 cells were injected subcutaneously into NPG mice and tumor volumes were measured at indicated dates. *n* = 5 mice. (**g**) Control shRNA or shRNAs targeting IL11 and SERPINE2 were transduced into SMMC7721 cells with a YTHDF2-knockdown (KD) background. Proliferative activity of indicated SMMC7721 cells was assessed by CCK8 (upper) or WST1 assay (lower). *n* = 5 biological replicates. (**h**) Numbers of endothelial tubes formed by HUVEC cocultured with indicated SMMC7721 cells. *n* = 3 biological replicates. (**i**) Indicated SMMC7721 cells were injected subcutaneously into NPG mice and tumor volumes were measured at indicated time points. *n* = 5 mice. (**j**) Immunohistological staining of IL-11 and SerpinE2 in human HCC tissue arrays divided as YTHDF2^Low^ and YTHDF2^High^ categories according to median integrated optical density (IOD) value of YTHDF2. *n* = 143 patients. Scale bar, 200 μm. Error bars indicate means ± SEM *P < 0.05, **P < 0.01, ***P < 0.001, ****P < 0.0001. *P*-values were determined by two tailed *t*-test

As a specific m^6^A ‘reader’ protein, YTHDF2 depends on the hydrophobic residues W432 and W486 in its carboxy-terminal YTH domain for selective recognition of m^6^A [17, 40]. To understand the molecular basis of our findings, we mutated the m^6^A recognition sites into W432A and W486A, which abrogate the specific binding affinity of YTHDF2 (Additional file 1: Figure S6A, B). A technical deviation is that the strain carrying a W432A mutation showed a little bit lower YTHDF2 protein level than wild-type control, but the W486A version was comparable to wild-type. In YTHDF2-deficient cells, re-expression of wild-type YTHDF2 but not the catalytically inactive mutant offset the acquired cancer-promoting inflammatory phenotypes (Fig. 5a, b), and reduced both phosphorylation of STAT3 and stabilization of IL11 and SERPINE2 mRNAs (Fig. 5c-e and Additional file 1: Figure S6C, D). In concert with this, redundant expression of catalytically dead YTHDF2 failed to inhibit in vivo tumor development (Fig. 5f and Additional file 1: Figure S6E). These results suggest that the m^6^A reader function is indispensable for the suppressive role of YTHDF2 in HCC.

To ascertain the importance of YTHDF2-IL11/SERPINE2 pathway in HCC development, we silenced IL11 or SERPINE2 in YTHDF2-deficient cells by using shRNAs. Removal of IL11 abrogated the growth competitiveness of YTHDF2-deficient SMMC7721 cells (Fig. 5g), while a SERPINE2 deficit attenuated the proangiogenic capacity of SMMC7721 cells (Fig. 5h), highlighting that IL11 and SERPINE2 are key targets of YTHDF2 in cancer-promoting inflammation. As anticipated, concomitant targeting of both IL11 and SERPINE2 counterbalanced the malignant capability endowed by YTHDF2 depletion (Fig. 5i and Additional file 1: Figure S6F). To examine this pathway in a clinical setting, we looked into human HCC tissues with relatively low or high YTHDF2 protein levels. As shown in Fig. 5j, IL-11 and Serpin E2 expression displayed negative correlations with YTHDF2.

### Hypoxia interrupts YTHDF2 expression in a HIF-2α-dependent manner

**Fig. 6:**
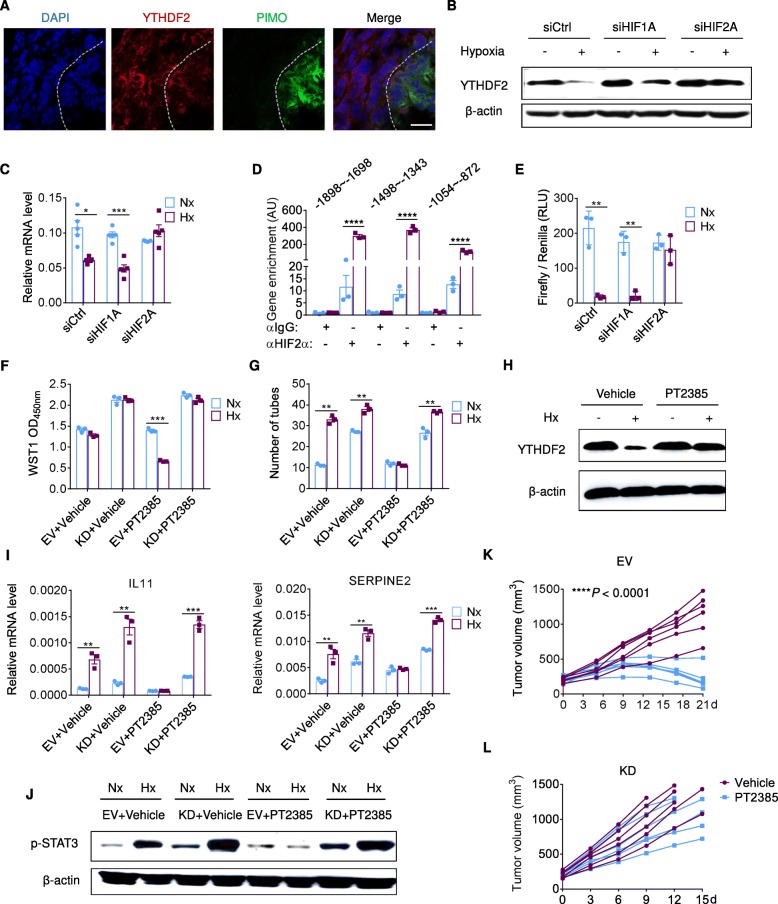
HIF-2α blockade contributed to YTHDF2-dependet HCC inhibition. (**a**) Immunofluorescence staining of YTHDF2 and pimonidazole (PIMO) in SMMC7721-derived mouse tumors. Hypoxic tumor areas were marked by PIMO staining. *n* = 3 biological replicates. Scale bar, 20 μm.(**b**) Immunoblot of YTHDF2 in SMMC7721 cells transduced with a control siRNA (siCtrl) or siRNAs targeting HIF-1/2α and grown for 24 h under Nx or Hx. *n* = 2 independent experiments. (**c**) RT-qPCR analysis of YTHDF2 in SMMC7721 cells expressing indicated siRNAs after 24 h of Nx or Hx exposure. (**d**) Enrichment of hypoxia-responsive elements (HREs) -containing fragments of human *Ythdf2* promoter in DNA-HIF-2α complex, as determined by ChIP-qPCR. *n* = 4 biological replicates. (**e**) *Ythdf2* promoter activity in SMMC7721 cells expressing indicated siRNAs, as quantified using luciferase assay. *Renilla* luciferase activity was normalized to firefly activity and presented as relative luciferase activity. *n* = 3 biological replicates. (**f**) Proliferative activity of indicated SMMC7721 cells treated with vehicle or PT2385 (10 μM), as assessed by WST1 assay. *n* = 3 biological replicates. (**g**) Numbers of endothelial tubes formed by HUVEC cocultured with indicated conditions. *n* = 3 biological replicates. (**h**, **j**) Immunoblot of YTHDF2 (**h**) and p-STAT3 (**j**) in SMMC7721 cells treated with vehicle or PT2385 (10 μM). *n* = 2 independent experiments. (**i**) RT-qPCR analysis of IL11 (left) and SERPINE2 (right) mRNA levels. *n* = 3 biological replicates. (**k**, l) NPG mice bearing SMMC7721-shCtrl (K) or SMMC7721-shYTHDF2 (L) cells were orally treated with Vehicle or PT2385 (20 mg/kg/d) after reaching an average tumor volume of 200 mm^3^. Tumor volumes were continuously measured at indicated dates of treatment. *n* = 6 mice. Error bars indicate means ± SEM *P < 0.05, **P < 0.01, ***P < 0.001, ****P < 0.0001. *P*-values were determined by two tailed *t*-test

Hypoxic tumor areas showed relatively low YTHDF2 expression in the xenograft mouse model (Fig. 6a), providing an in vivo evidence for hypoxia-mediated YTHDF2 reduction. A recent report showed that HIF-1α was responsible for this negative regulation [19]. However, when we sought to block the hypoxia signaling in vitro, only targeting HIF-2α completely rescued YTHDF2 expression in hypoxic cells (Additional file 1: Figure S7A-C and Fig. 6b, c). Containing the hypoxia-responsive elements (HREs, *CGTG*), the *Ythdf2* promoter was assessed through chromatin immunoprecipitation (ChIP)-qPCR and demonstrated HIF-2α binding (Fig. 6d). Additionally, we inserted the *Ythdf2* promoter into a pGL4 luciferase reporter plasmid. While *Ythdf2* promoter activity was decreased upon hypoxia, this was reversed by a HIF-2α (but not HIF-1α) siRNA (Fig. 6e and Additional file 1: Figure S7D). In human tissues, HIF-2α was detected in the cytoplasm of non-tumor hepatocytes (Additional file 1: Fig. S7E), whereas it was aggregated in the nucleus of tumor cells, where it could regulate gene transcription.

Since HIF-2α often terminates further expansion of hypoxic tumors, a small molecule (PT2385) selectively targeting HIF-2α has been developed to treat renal cell carcinoma and hepatocellular carcinoma [29, 30]. We tested PT2385 in treating HCC cells in vitro and in vivo, and found it exhibited favorable therapeutic effects (Fig. 6f, g, k). PT2385 rescued YTHDF2 expression (Fig. 6h) but unaffected YTHDF2 localization in hypoxic cells (data not shown), accompanied by neutralization of IL11 and SERPINE2 expression, as well as STAT3 phosphorylation (Fig. 6i, j). Nonetheless, PT2385 administration to the mice bearing YTHDF2-deficient tumors failed therapeutically (Fig. 6l), which was indicative of an indispensable role of YTHDF2 in treating hypoxic HCC.

## Discussion

**Fig. 7:**
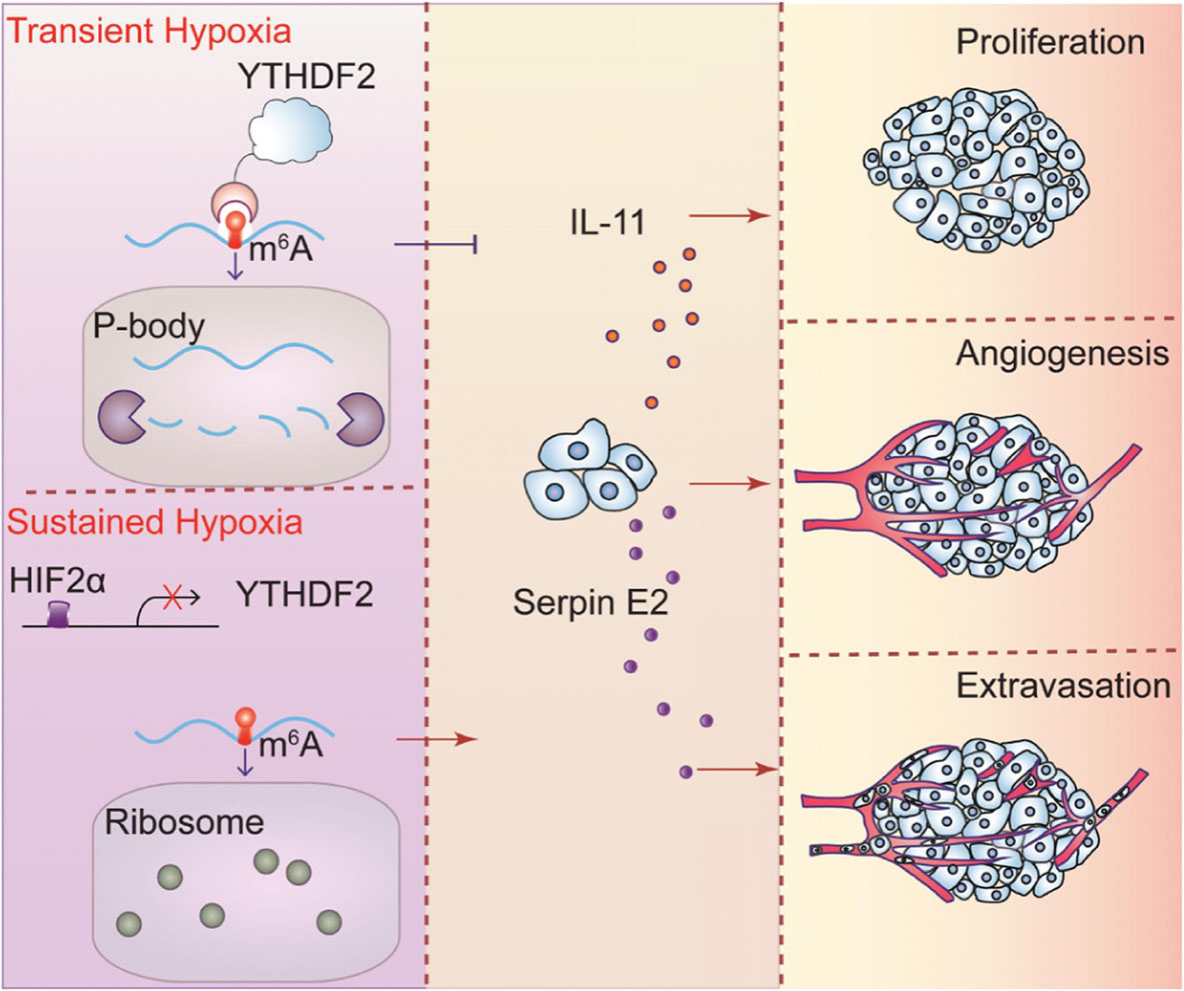
Schematic model showing how hypoxia perturbs YTHDF2 to promote cancer-associated inflammation. P-body, processing body

This study specified the suppressive potential of YTHDF2 in cancer-promoting inflammation, and established a context of metabolic-epigenetic regulation where hypoxia imposes a cancer-specific procedure of m^6^A-mRNA editing. Importantly, restoring YTHDF2 expression defined m^6^A decoration as a tumor suppressive mechanism. Despite the sensitivity of destabilizing IL11 and SERPINE2 mRNAs in response to tumor hypoxia, YTHDF2 expression is readily silenced in the presence of active HIF-2α (Fig. 7). In accordance, HIF-2α blockade improves YTHDF2-mediated suppression on m^6^A-mRNA stability and inflammatory cancer behaviors, leveraging this metabolic-epigenetic axis toward therapeutic opportunities in human HCC.

Unlike methylation-related inhibition of tumor suppressor gene promoters, the epigenomic hallmarks and functional relevance for m^6^A mRNA decoration have not been fundamentally defined in human cancers. Here and elsewhere [13] it has been shown that m^6^A labeling is blunted in total RNAs of human HCC tissues, and yet tumor mRNAs exhibit m^6^A hypermethylation. This could be explained by the possibility of m^6^A hypomethylation in other RNAs, since m^6^A was also found in transfer RNA (tRNA), ribosomal RNA (rRNA), small nuclear RNA (snRNA) and long non-coding RNA (lncRNA) [41]. Here we attach importance to abnormal mRNA methylation-related gene expression and biological functions in human HCC. To map its epitranscriptomic landscape, we took advantage of m^6^A- and mRNA-seq methods and thus quantitatively compared transcriptome-wide changes between tumor and non-tumor tissues. Typically, the majority of m^6^A-labelled mRNAs were regulated in a ‘hyper-up’ manner, which assembled a variety of oncogenic pathways. Interestingly, introducing HCC cells to hypoxia enabled this epitranscriptomic transformation in vitro, affecting common oncogenes such as those involved in ‘serine-type endopeptidase inhibitor activity’. Putatively, the ‘hyper-up’ feature is caused, at least in part, by hypoxia-induced YTHDF2 reduction. It has been widely accepted that steady-state levels of most transcripts decrease in the presence of m^6^A modification. Nonetheless, a deficit in YTHDF2 expression may shift m^6^A-editing into a cancer-specific mode. This notion is also supported by the recent observations that certain m^6^A-labelled genes become more stable in cancers [4, 42]. Taken together, our findings suggest hypoxia acts to shape the distinct m^6^A-mRNA landscape in human HCC.

Most HCC patients share a non-resolved inflammatory condition, owing to a history of chronic liver diseases with viral, fatty or other causes. In an autocrine or paracrine fashion, HCC cells receive the malignant signals from pro-inflammatory cytokines, which boost tumor and vascular proliferation and metastatic invasion. Blockade of oncogenic and angiogenic kinases or downstream STAT3 can retard primary tumor growth, which provides a rationale for many mainstream clinical therapeutics [43]. Here we identify YTHDF2 as a novel tumor suppressor resolving cancer-promoting inflammation. Mechanistically, YTHDF2 inhibited STAT3 phosphorylation and tumor growth by degrading IL11 mRNA, which encodes the dominant IL-6 family cytokine that endows gastrointestinal cancers with proliferative and invasive capacity [27, 36]. The recent report showed that YTHDF2 could suppress EGFR phosphorylation [19], suggesting the substrate of YTHDF2 is in the upstream of EGFR but not EGFR itself.

Given the hypervascularity of HCC or metastatic liver cancer, anti-angiogenic therapy has long been considered unique; however, accumulating evidence in other cancer types have yielded insights into the necessity of both angiogenic and nonangiogenic mechanisms for targeting tumor vasculature. The latter accounts for the acquired resistance of anti-angiogenic therapy and metastatic dissemination of cancer cells, including but not limited to vascular co-option and mimicry [27, 44]. In this context, we unexpectedly found that YTHDF2 resisted either tumor angiogenesis or vascular mimicry by targeting SERPINE2. Serpins, or serine-type endopeptidase inhibitors, are a superfamily of secretory proteins acting as anti-coagulant and pro-inflammatory factors, which frequently display m^6^A-mRNA hypermethylation and gene overexpression in human HCC. Plasminogen activator (PA) inhibitory serpins have been shown to promote tumor growth and initiate metastasis by fostering angiogenesis and vascular co-option [37, 38]. As previously reported, the autocrine of serpin E2 of breast cancer cells enabled vascular mimicry and drove metastatic extravasation [28]. We show that YTHDF2 is required for suppressing tumor angiogenesis in vitro and in vivo, and restricting formation of vascular mimicry in the metastatic MHCC97H tumors. Strikingly, YTHDF2 also benefited tumor vessel normalization [35], as characterized by a positive correlation to pericyte coverage and an inverse correlation to vascular permeability. Therefore, these results indicated that YTHDF2 provided the ideal molecular basis to control HCC by pleiotropic targeting of cancer-promoting inflammation.

Under normoxia, introduction of exogenous YTHDF2 in HCC cells could slightly mediate IL11 and SERPINE2 mRNA decay but did not correspondently disrupt the 3’UTR reporter activity in the presence of m^6^A motifs. A possible explanation is that the simplified 3’UTR constructs could not faithfully reflect the interaction between substrate mRNAs and YTHDF2. By contrast, endogenous YTHDF2 seemed more functional than the exogenous version, hence the expression of IL11 and SERPINE2 mRNAs were significantly upregulated when knocking down YTHDF2. Importantly, YTHDF2 function was reinforced by oxygen deficit, owing to its increased distribution in processing bodies.

Hypoxia is a key driver of tumor growth, angiogenesis and metastasis, and also a mediator preconditioning re-vascularization and tumor dissemination post therapeutic vascular regression [34, 44]. Clinically, hypoxia-induced HIF stabilization was associated with poor patient survival in a variety of cancer types [34]. Despite a small portion of overlapping target genes, HIF-1α and HIF-2α often exert different functions at different stage of cancer development. For instance, HIF-1α and HIF-2α play complementary roles during early angiogenesis, whereas vascular remodeling are mainly HIF-2α-driven [45]. In certain types of cancer, a HIF-1α-to-HIF-2α switch evolutionarily emerges to enhance aggressive tumor growth and invasion [46]. Of note, abnormal HIF-2α expression could even occur under normoxic conditions [47], which we also detected in HCC cell lines. Since non-tumor liver tissues accumulated abundant HIF-2α in the cytoplasm of hepatocytes, it is highly suggestive for its potential cytosolic function. In line with increased nuclear expression in tumor regions, we uncover its association with *Ythdf2* gene promoter and transcriptional suppression. Intriguingly, recent work suggested that the first-line anti-HCC drug sorafenib induced HIF-2α expression, whereas treating with the HIF-2 antagonist PT2385 significantly reversed sorafenib efficiency [30]. Here, we demonstrate that PT2385 rescues and requires YTHDF2 expression to exert its therapeutic effect in a HCC xenograft model. Besides transcriptional regulation, hypoxic onset allows translocation of cytosolic YTHDF2 protein to processing bodies through an unknown mechanism. In concert, PT2385 upregulates YTHDF2 expression without changing its cytosolic distribution, which optimizes YTHDF2 function in hypoxic cancer.

## Conclusions

The m^6^A ‘reader’ YTHDF2 acts as a mRNA-processing enzyme, potentially reprogramming the epitranscriptome in response to hypoxia, a frequent feature in human HCC. Our results indicate that YTHDF2 represses both tumor cells and tumor vasculature by processing IL11 and SERPINE2 mRNAs to decay. Indeed, HIF-2α-mediated inhibition of YTHDF2 in HCC cells provides proof of concept that hypoxia adapts cancer epigenetics for aggressiveness, which is due, at least in part, to the stabilization of m^6^A-containing oncogene mRNAs. Overall, these findings highlight profound implications for understanding and targeting the m^6^A-methylome in human liver cancer.

## Materials and methods

### Patient specimens

Tumor tissues and matched adjacent non-tumor tissues were obtained from liver cancer patients who underwent liver resection at the Department of Liver Surgery, and diagnosed as HCC by the Department of Pathology, Renji Hospital, School of Medicine, Shanghai Jiaotong University. All samples were collected with informed consent, and the experiments were approved by the ethical review committee of the World Health Organization Collaborating Center for Research in Human Production (authorized by the Shanghai Municipal Government). A total number of 200 patients enrolled from 2006 to 2012 were all treatment naïve before surgery, and were routinely followed up after curative liver resection. Portions of surgically resected tissues were immediately transferred to liquid nitrogen until RNA or protein extraction. Paraffin-embedded tumors were processed into tissue microarrays for histological staining and prognostic analysis. Detailed clinicopathologic features were summarized in Additional file 2: Table S1.

### m^6^A immunoblotting

Poly(A)^+^ mRNA samples were denatured at 65 °C for 5 min in 3 sample volumes of RNA incubation buffer. An equal volume of chilled 20 × SSC buffer (Sigma-Aldrich) was then added before samples were spotted on the nitrocellulose membrane. After UV crosslinking for 10 min, the membrane was washed with 1 x PBST buffer, blocked with 5% non-fat milk and incubated with anti-m^6^A antibody (1:2000, Synaptic Systems) overnight at 4 °C. Then HRP-conjugated goat anti-rabbit IgG (Jackson Immuno Research Laboratories) was added to the blots for 1 h at room temperature and the membrane was developed with a ChemiDoc XRS system (Bio-Rad).

### LC-MS/MS quantification of m^6^A

100–200 ng of mRNA was digested by nuclease P1 (2 U) in 25 μl of buffer containing 25 mM NaCl, and 2.5 mM ZnCl_2_ at 42 °C for 2 h, followed by the addition of NH_4_HCO_3_ (1 M, 3 μl) and alkaline phosphatase (0.5 U) and incubation at 37 °C for 2 h. The sample was then filtered (0.22 μm pore size, 4 mm diameter, Millipore), and 5 μl of the solution was injected into the LC-MS/MS. The nucleosides were separated by reverse- phase ultra-performance liquid chromatography on a C18 column with online mass spectrometry detection using an Agilent 6410 QQQ triple-quadrupole LC mass spectrometer in positive electrospray ionization mode. The nucleosides were quantified by using the nucleoside-to-base ion mass transitions of 282 to 150 (m^6^A) and 268 to 136 (A). Quantification was carried out by comparison with a standard curve obtained from pure nucleoside standards run with the same batch of samples. The m^6^A/A ratio was calculated based on the calibrated concentrations.

### m^6^A-IP (MeRIP)

Total RNA was extracted from human HCC tissues or SMMC7721 cells with TRIzol (Invitrogen), then polyadenylated RNA was enriched using an Oligotex® mRNA purification kit (Qiagen). In particular, additional DNase I digestion was applied to all samples to avoid DNA contamination. RNA fragmentation, m^6^A-IP and library preparation were proceeded according to previously published protocols [41]. Sequencing was conducted at the University of Chicago Genomics Facility on an Illumina HiSeq2500 machine in single-read mode with 50 base pairs per read. Enrichment of m^6^A-containing transcript segments was also analyzed through RT-qPCR. Primers targeting m^6^A-enriched regions of IL11 and SERPINE2 are listed: IL11_F, TCCAACAGTGAGGGTTAAGCAA; IL11_R, CCCTGAATGACAGTCCCTGC; SERPINE2_F, GGCCTCATGACAACATCGTG; SERPINE2_R, CGAGCTGCTTCTTGGTCCTG.

### Gene set enrichment analysis (GSEA)

RNA-seq read counts for 14 genes that make up the hypoxia metagene signature (ALDOA, MIF, TUBB6, P4HA1, SLC2A1, PGAM1, ENO1, LDHA, CDKN3, TPI1, NDRG1, VEGFA, ACOT7 and ADM) [32] were analyzed with GSEA software v.2.0, which is available from the Broad Institute ().

### Animal procedures and models

A number of 5 × 10^6^ SMMC7721 or MHCC97H cells re-suspended in 100 μl of PBS were subcutaneously injected into the right flank of 6-week old male NCG mice (NOD-*Prkdc*^scid^ *Il2rg*^null^) (Model Animal Research Center of Nanjing University). Tumor growth was monitored by caliper rule every 3–5 days. Primary tumor volume was measured using the formula: Volume = (Length × Width ^2^)/2. The mice were euthanized when the tumor length reached 15 mm or at the indicated date post tumor injection. Tumors and lungs were harvested for further histological study. For HIF-2 antagonism, PT2385 (c) was suspended in saline with 0.5% sodium carboxymethyl cellulose, 2.5% Tween 80 and 2.5% dimethyl sulfoxide. NPG mice were randomly distributed into 2 groups when the subcutaneous tumors reached an average size of 200 mm^3^, and administered vehicle or PT2385 (20 mg/kg o.p.d.) by gavage until endpoint (when the tumor size in control group reached 15 mm).

*Ythdf2*^F/F^ mice in C57BL/6 background were described elsewhere [48]. For liver-specific disruption, *Ythdf2*^F/F^ mice were crossed with mice harboring the Cre recombinase under control of the albumin promoter to obtain the *Ythdf2*^LKO^ strain. Male *Ythdf2*^F/F^ and *Ythdf2*^LKO^ littermates were injected intraperitoneally with a single dose of 25 mg/kg diethylnitrosamine (DEN) (Sigma-Aldrich) on postnatal day 14, and 6 weeks later administrated CCl_4_ (1 ml/kg) once per week for 12 weeks. Mice were euthanized at 8.5-month old, and the livers and lungs were harvested for tumor assessment and histological experiments. The primers used for genotyping are listed: 5’*Ythdf2*_F, GCTTGCCTGCTACATAGTGAGA; 5’*Ythdf2*_R, AACTGAACTGCTTAACCTTCTGG; 3’*Ythdf2*_F, GAACGGTATTGTCGGTATTGTCA; 3’*Ythdf2*_R, AGACCACTCCAACACAGAACTT.

All mice were maintained under specific pathogen-free (SPF) conditions, on a 12 h light-dark cycle. All mouse experiments were approved by the Shanghai Administrative Committee for Laboratory Animals.

### YTHDF2-RIP

The procedure was adapted from the previous protocol [49] and was described in our recent work [16]. Briefly, a total of 5 × 10^7^ SMMC7721 cells expressing Flag-tagged YTHDF2 were collected and lysed in a buffer containing 150 mM KCl, 10 mM HEPES (pH 7.6), 2 mM EDTA, 0.5% CA-630, 0.5 mM DTT, 1:100 protease inhibitor cocktail, and 400 U/ml RNase inhibitor. Cell lysate was incubated with Flag M2 beads at 4 °C for 4 h. Then the beads were washed with NT2 buffer and incubated in elution solution containing Flag peptide (Sigma-Aldrich). After washing, bound RNAs were extracted using TRIzol reagent and analyzed by RT-qPCR.

### RNA lifetime assay

SMMC7721 cells were exposed to hypoxia or normoxia, and 12 h later actinomycin D (MCE) was added at a concentration of 5 mg/ml. At the indicated time points, the cells were trypsinized and collected for RNA purification. RNA quantities were determined by RT-qPCR. The degradation rate of RNA (k) was calculated using the equation:

$$ \frac{N_t}{N_0}={e}^{- kt} $$

where *t* is the transcription inhibition time, *k* is the degradation rate, and *N*_*t*_ and *N*_*0*_ are the RNA quantities at time t and time 0. The RNA lifetime (*t*_*1/2*_) can be calculated from the degradation rate as follows:

$$ {t}_{\frac{1}{2}}=\frac{\mathit{\ln}2}{k} $$

### Statistical analysis

At least three biological replicates were used in each experiment unless otherwise stated. Data were analyzed with GrapPad Prism 7 and were presented as the mean ± standard error of the mean (SEM). Comparisons between two groups were assessed using unpaired or paired (for matched comparisons) two-tailed Student’s t-test, or non-parametric Mann-Whitney U-test. Multiple comparisons were assessed by one-way ANOVA. Survival rates were compared using the log-rank test. Pearson correlation coefficients (*r*) were calculated to assess correlation and statistical significance was assessed by a two-tailed t-test of *r* = 0. The statistical significance of clinicopathological differences among HCC patients were assessed by *χ*^2^-test.

### Supplementary materials and methods

More detailed materials and methods including Cell culture, Constructs and transfections, Immunohistochemistry, Immunoblotting, Real-time quantitative PCR, Immunofluorescence, Cell proliferation, migration and mammosphere assay, Tube formation assay, Enzyme-linked immunosorbent assay, Luciferase reporter assay, Chromatin immunoprecipitation, m^6^A-seq data analyses are provided in Additional file 5.

## Supplementary information

**Additional file 1:** Supplementary Figures. **Figure S1.** Identification of a “hyper-up” pattern in m6A-epitranscriptome of human HCC. **Figure S2.** Expression of m6A modulators in human HCC tissues and hypoxic HCC cell lines. **Figure S3.** YTHDF2 deficiency enhances proliferative and proangiogenic functions of HCC cells. **Figure S4.** YTHDF2 inhibits tumor growth and vasculature remodeling. **Figure S5.** YTHDF2 deficiency upregulates IL-11 and Serpin E2 expression in HCC cells. **Figure S6.** YTHDF2 requires its recognitive function to degrade IL11 and SERPINE2 mRNAs. **Figure S7.** HIF-2α transcriptionally inhibits YTHDF2 expression in HCC cells. **Figure S8.** Unprocessed original scans of blots.

**Additional file 2: Table S1.** Clinicopathological information of 200 HCC patients.

**Additional file 3:** **Table S2.** Upregulated genes in YTHDF2-deficient SMMC7721 cells.

**Additional file 4: Table S3.** Downregulated genes in YTHDF2-overexpressed SMMC7721 cells.

**Additional file 5:** Supplementary Information.

## Data Availability

The RNA-seq and MeRIP-seq data generated in this study have been deposited in the GEO database under the accession number GSE120860, GSE120659 and GSE120611. The transcriptomic profiles of SMMC7721 cells with diverse YTHDF2 expression can be found in Additional file 3: Table S2 and Additional file 4: Table S3. All the other data generated or analyzed during this study are included in the article and Additional file 5.

## Abbreviations

ALKBH5: a-ketoglutarate-dependent dioxygenase alkB homolog 5
CDS: Coding regions
FTO: Fat mass-and obesity-associated protein
HCC: Hepatocellular carcinoma
HIF: Hypoxia-inducible factor-2α
IL-11: Interleukin 11
m^6^A: *N*^6^-methyladenosine
METTL3: Methyltransferase-like 3
PA: Plasminogen activator
RIP: RNA-immunoprecipitation
Serpin: Serine-type endopeptidase inhibitor
UTR: Untranslated regions
YTHDF2: YTH domain family 2
