# Correction to: YTHDF2 reduction fuels inflammation and vascular abnormalization in hepatocellular carcinoma

**DOI:** 10.1186/s12943-020-01257-8

**Published:** 2020-09-04

**Authors:** Jiajie Hou, He Zhang, Jun Liu, Zhenjun Zhao, Jianye Wang, Zhike Lu, Bian Hu, Jiankui Zhou, Zhicong Zhao, Mingxuan Feng, Haiyan Zhang, Bin Shen, Xingxu Huang, Beicheng Sun, Mark J. Smyth, Chuan He, Qiang Xia

**Affiliations:** 1 Department of Liver Surgery, Renji Hospital, School of Medicine, Shanghai Jiaotong University, Shanghai, 200127 China; 2 Department of Hepatobiliary Surgery, The Affiliated Drum Tower Hospital of Nanjing University Medical School, Nanjing, 210093 China; 3 Department of Hepatobiliary Surgery, Sun Yat-sen University Cancer Center, Guangzhou, 510060 China; 4 State Key Laboratory of Oncology in South China, Sun Yat-sen University Cancer Center, Guangzhou, 510060 China; 5 Department of Surgery, The University of Hong Kong-Shenzhen Hospital, Shenzhen, 518053 China; 6 Department of Chemistry, Department of Biochemistry and Molecular Biology, Institute for Biophysical Dynamics, University of Chicago, Chicago, IL 60637 USA; 7 School of Life Science and Technology, ShanghaiTech University, Shanghai, 201210 China; 8 Immunology of Cancer and Infection Laboratory, QIMR Berghofer Medical Research Institute, Herston, Queensland 4006 Australia; 9 Key Laboratory of Reproductive Medicine, Department of Histology and Embryology, Nanjing Medical University, Nanjing, 211166 China; 10 Howard Hughes Medical Institute, University of Chicago, Chicago, IL 60637 USA

**Correction to: Mol Cancer 18, 163 (2019)**

**https://doi.org/10.1186/s12943-019-1082-3**

Following the publication of the original article [1], the author list lacked a co-author’s name: Mark J. Smyth, and his affiliation is QIMR Berghofer Medical Research Institute. The correct author list should be “Jiajie Hou; He Zhang; Jun Liu; Zhenjun Zhao; Jianye Wang; Zhike Lu; Bian Hu; Jiankui Zhou; Zhicong Zhao; Mingxuan Feng; Haiyan Zhang; Bin Shen; Xingxu Huang; Beicheng Sun; Mark J. Smyth; Chuan He; Qiang Xia”. The error has been corrected in the HTML and PDF version of this article.
