# Supplementary material for: YTHDF2 reduction fuels inflammation and vascular abnormalization in hepatocellular carcinoma
Source: Mol Cancer. 2019 Nov 18;18:163. doi: 10.1186/s12943-019-1082-3 (PMC6859620; doi:10.1186/s12943-019-1082-3)

Figure S1

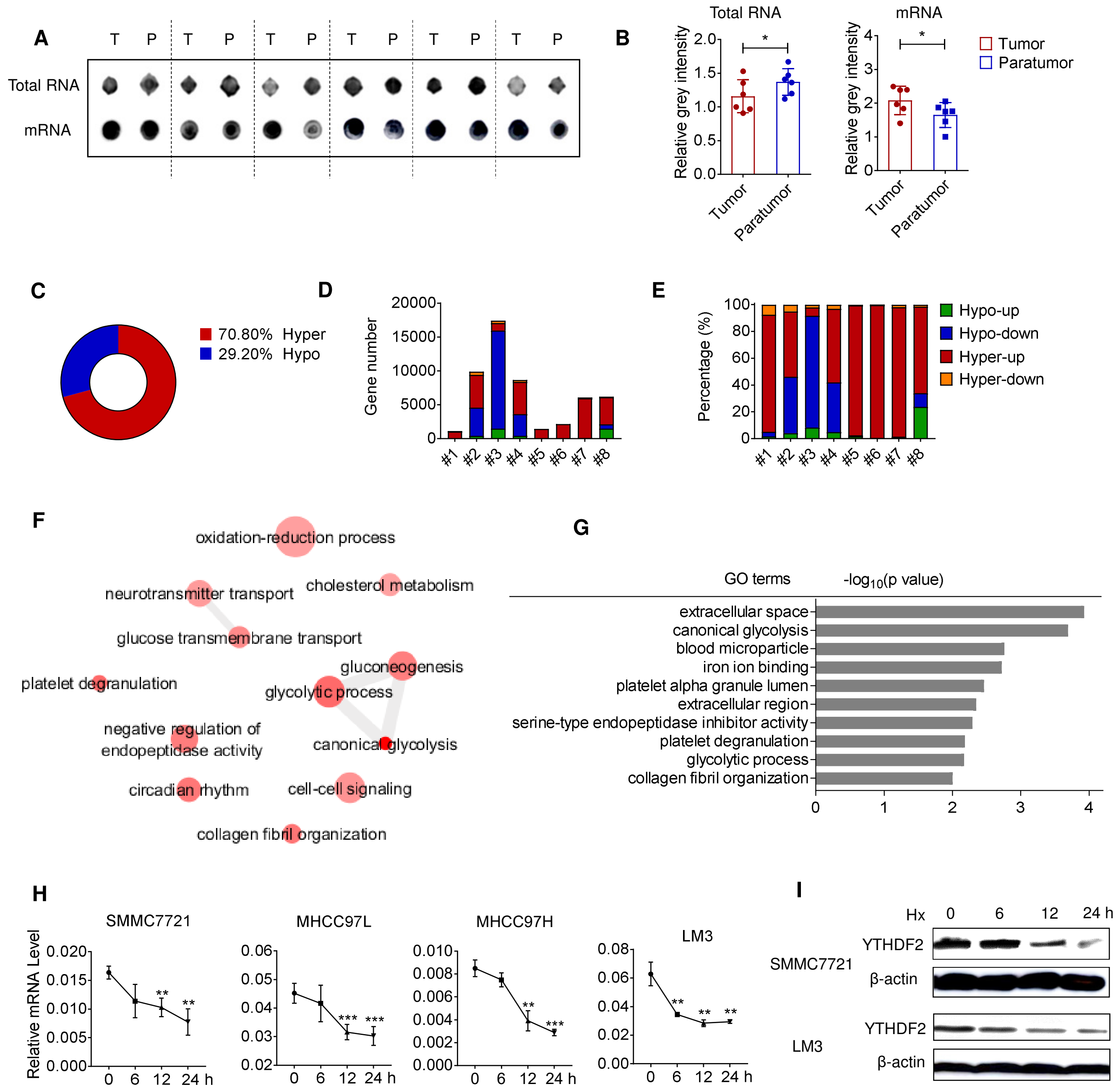
A
T P T P T P T P T P T P
Total RNA
mRNA
B
Total RNA
mRNA
Relative grey intensity
Tumor
Paratumor
C
70.80% Hyper
29.20% Hypo
D
Gene number
E
Percentage (%)
Hypo-up
Hypo-down
Hyper-up
Hyper-down
F
oxidation-reduction process
neurotransmitter transport
cholesterol metabolism
glucose transmembrane transport
gluconeogenesis
platelet degranulation
glycolytic process
negative regulation of endopeptidase activity
canonical glycolysis
circadian rhythm
cell-cell signaling
collagen fibril organization
G
GO terms
-log10(p value)
extracellular space
canonical glycolysis
blood microparticle
iron ion binding
platelet alpha granule lumen
extracellular region
serine-type endopeptidase inhibitor activity
platelet degranulation
glycolytic process
collagen fibril organization
H
SMMC7721
MHCC97L
MHCC97H
LM3
Relative mRNA Level
0 6 12 24 h
I
Hx 0 6 12 24 h
SMMC7721
LM3
YTHDF2
β-actin

Figure S2

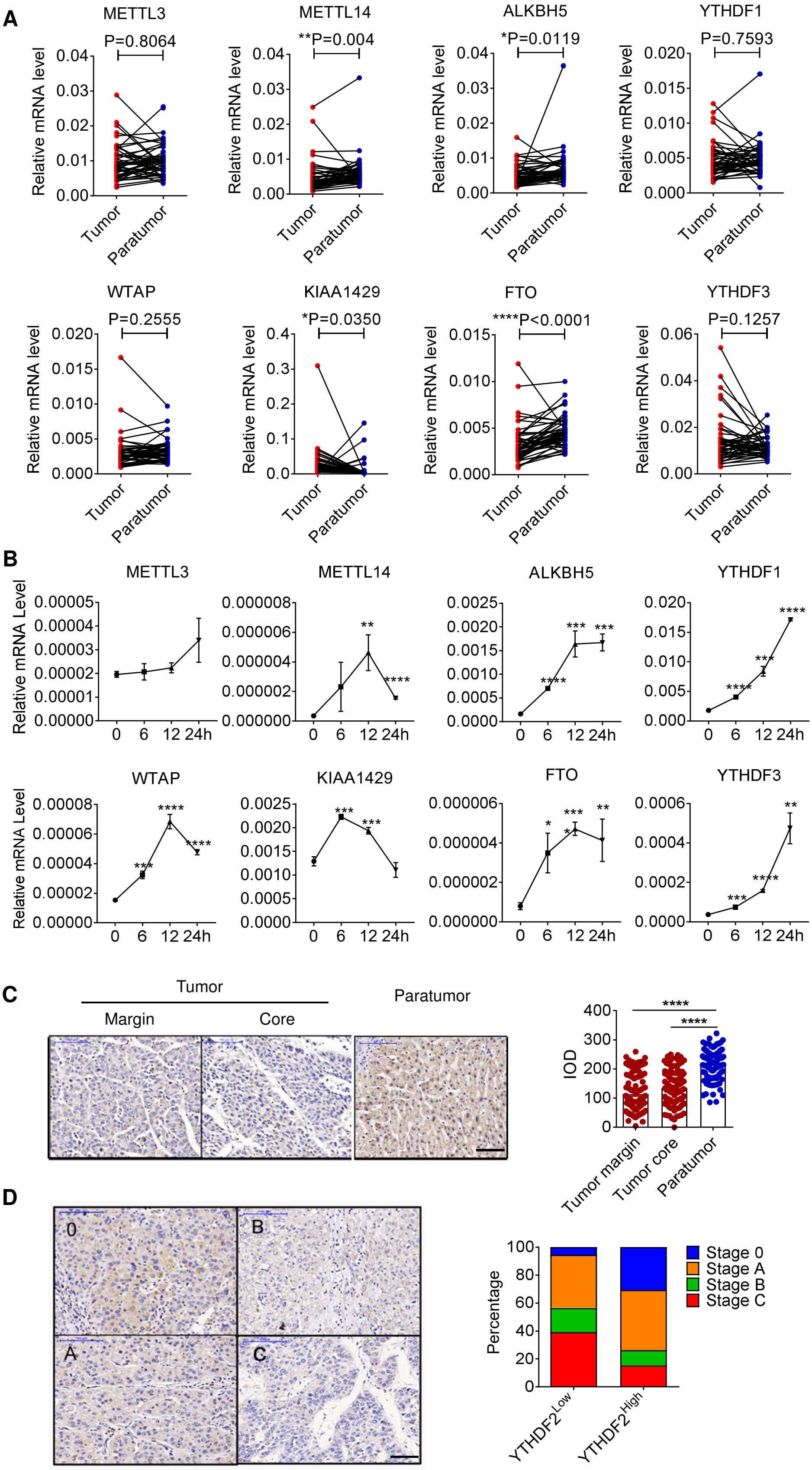
A
METTL3
P=0.8064
METTL14
**P=0.004
ALKBH5
*P=0.0119
YTHDF1
P=0.7593
WTAP
P=0.2555
KIAA1429
*P=0.0350
FTO
****P<0.0001
YTHDF3
P=0.1257
Relative mRNA level
Tumor
Paratumor
B
METTL3
METTL14
ALKBH5
YTHDF1
WTAP
KIAA1429
FTO
YTHDF3
Relative mRNA Level
0 6 12 24h
C
Tumor
Margin
Core
Paratumor
IOD
Tumor margin
Tumor core
Paratumor
D
0
A
B
C
Percentage
Stage 0
Stage A
Stage B
Stage C
YTHDF2Low
YTHDF2High

Figure S3

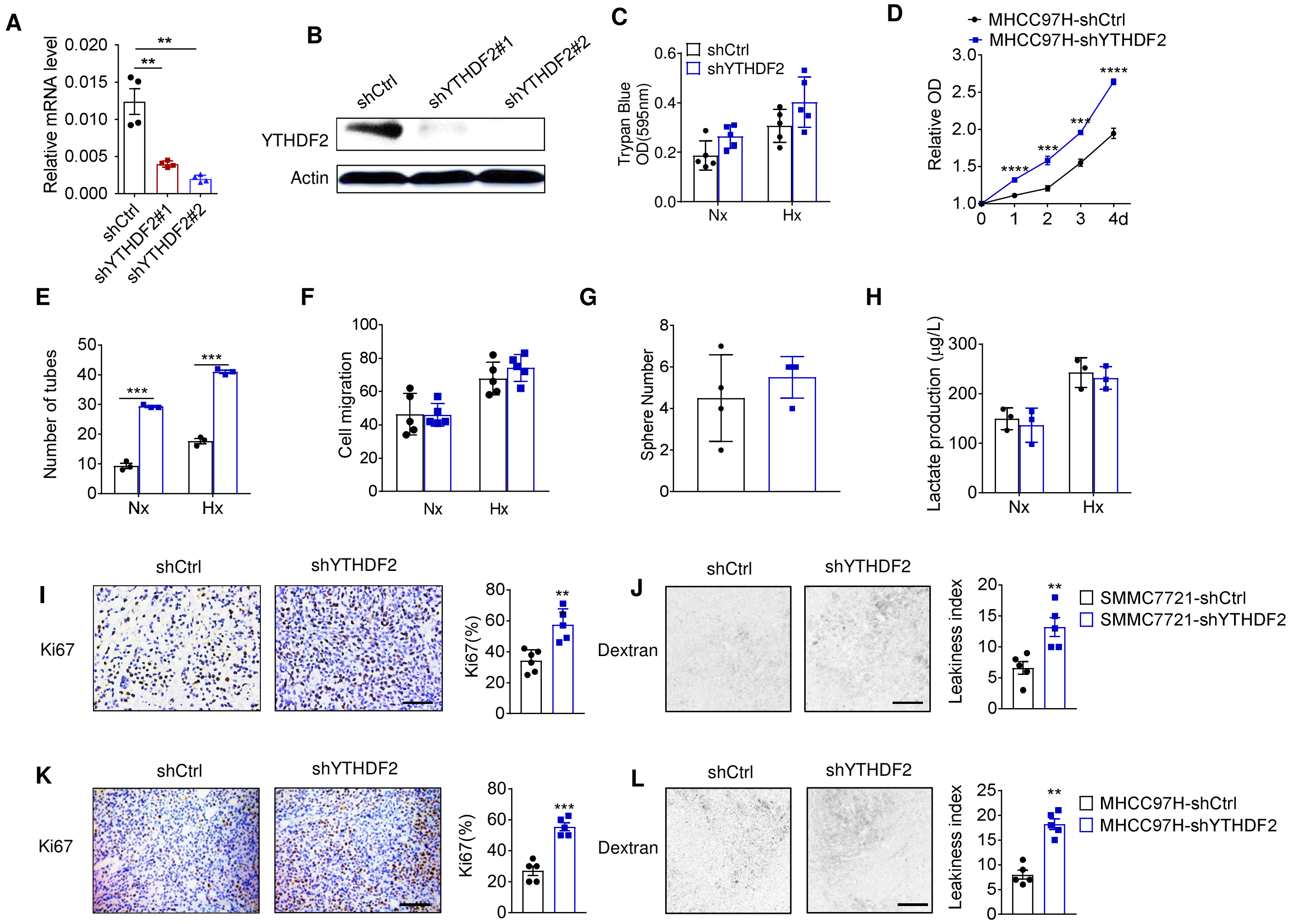

A
Relative mRNA level
shCtrl
shYTHDF2#1
shYTHDF2#2
B
YTHDF2
Actin
C
shCtrl
shYTHDF2
Trypan Blue OD(595nm)
Nx
Hx
D
MHCC97H-shCtrl
MHCC97H-shYTHDF2
Relative OD
E
Number of tubes
F
Cell migration
G
Sphere Number
H
Lactate production (μg/L)
I
Ki67
shCtrl
shYTHDF2
Ki67(%)
J
Dextran
Leakiness index
SMMC7721-shCtrl
SMMC7721-shYTHDF2
K
L
MHCC97H-shCtrl
MHCC97H-shYTHDF2

Figure S4

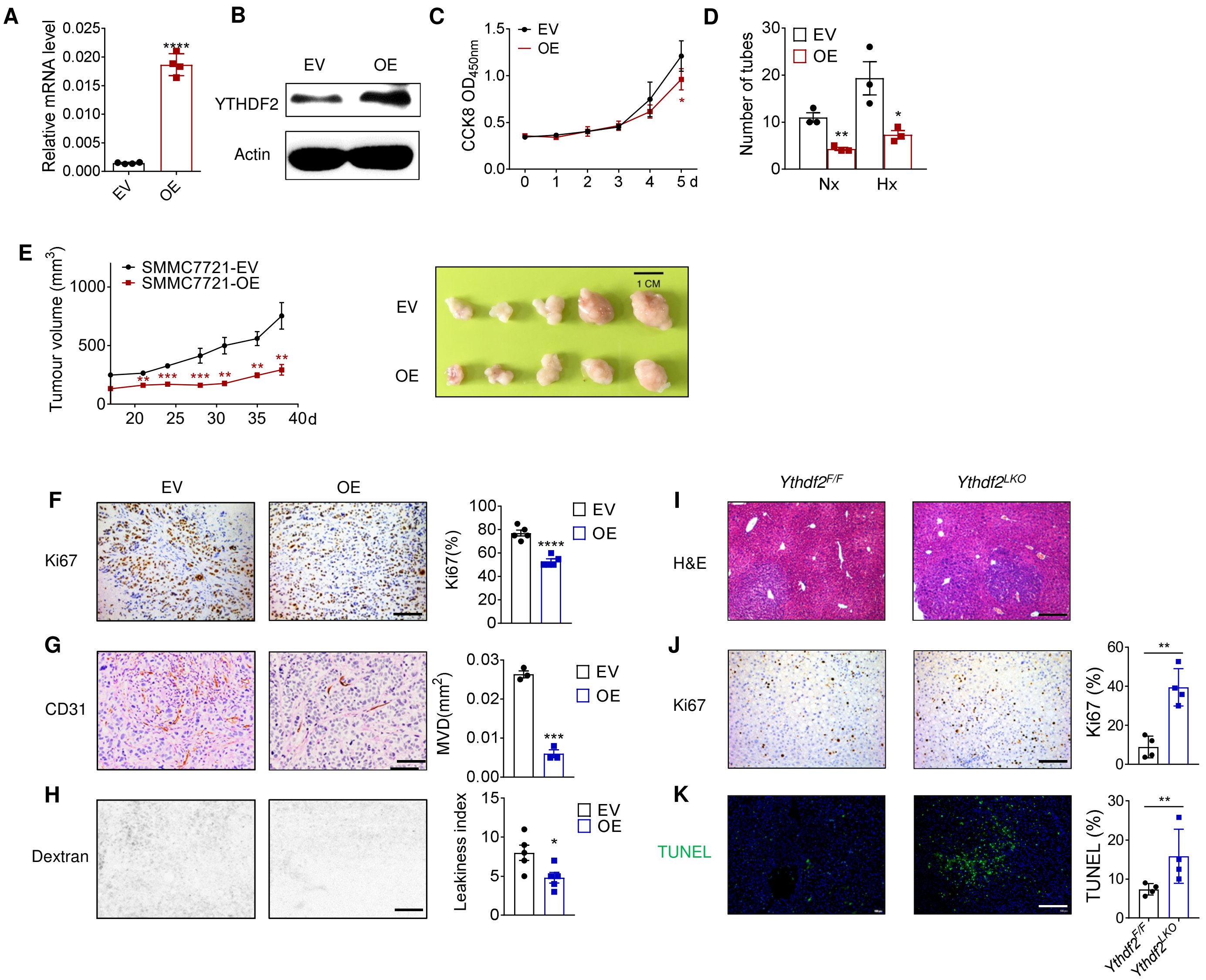

A
Relative mRNA level
0.025
0.020
0.015
0.010
0.005
0.000
****
EV
OE
B
EV
OE
YTHDF2
Actin
C
CCK8 OD450nm
EV
OE
1.5
1.0
0.5
0.0
0 1 2 3 4 5 d
*
D
Number of tubes
30
20
10
0
EV
OE
**
*
Nx
Hx
E
Tumour volume (mm3)
SMMC7721-EV
SMMC7721-OE
1000
500
0
20 25 30 35 40d
** *** *** ** ** **
EV
OE
1 CM
F
EV
OE
Ki67
Ki67(%)
100
80
60
40
20
0
****
EV
OE
G
CD31
MVD(mm2)
0.03
0.02
0.01
0.00
***
EV
OE
H
Dextran
Leakiness index
15
10
5
0
*
EV
OE
I
Ythdf2F/F
Ythdf2LKO
H&E
J
Ki67
Ki67 (%)
60
40
20
0
**
K
TUNEL
TUNEL (%)
30
20
10
0
**
Ythdf2F/F
Ythdf2LKO

Figure S5

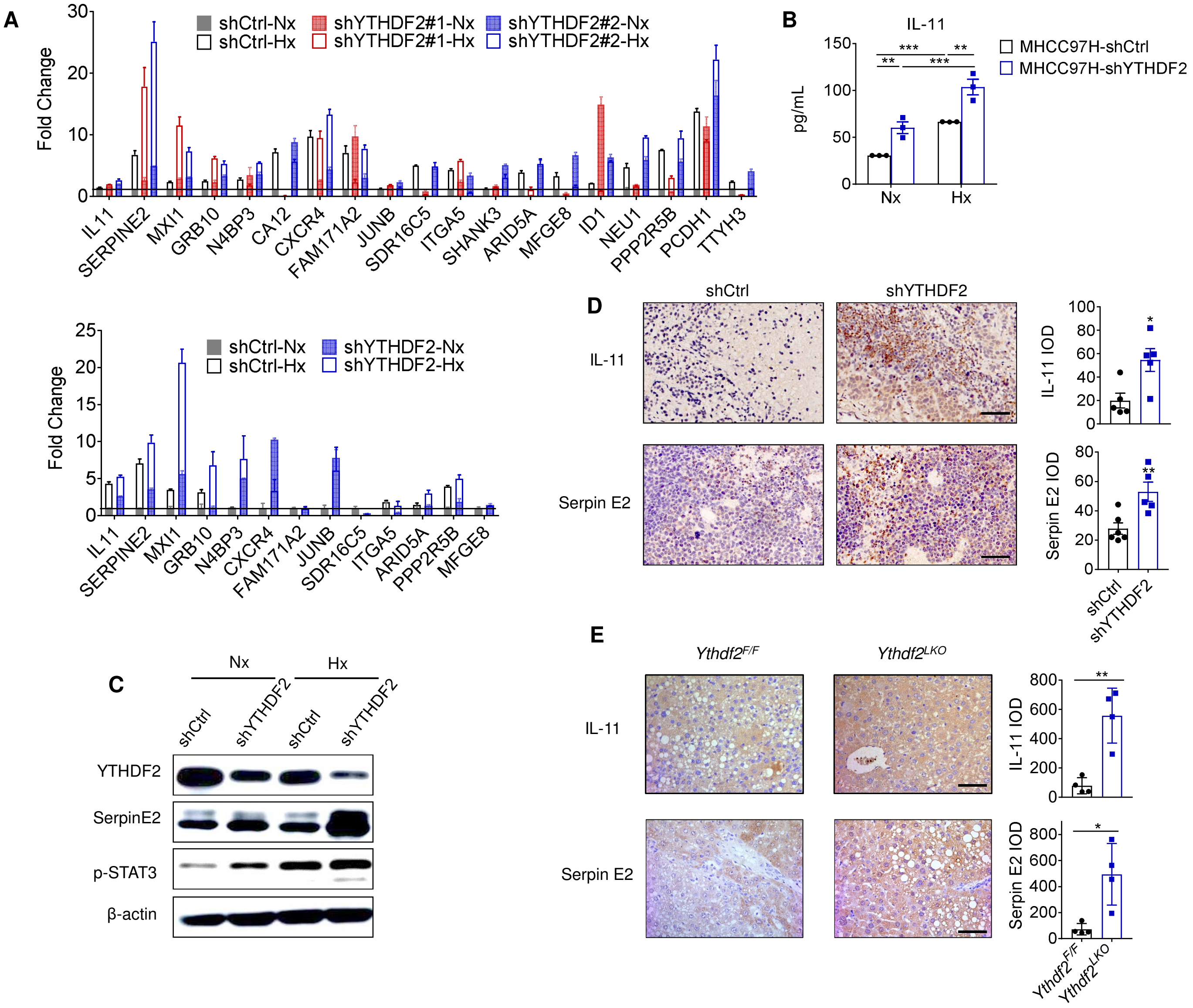
A
Fold Change
shCtrl-Nx
shCtrl-Hx
shYTHDF2#1-Nx
shYTHDF2#1-Hx
shYTHDF2#2-Nx
shYTHDF2#2-Hx
IL11
SERPINE2
MXI1
GRB10
N4BP3
CA12
CXCR4
FAM171A2
JUNB
SDR16C5
ITGA5
SHANK3
ARID5A
MFGE8
ID1
NEU1
PPP2R5B
PCDH1
TTYH3
shYTHDF2-Nx
shYTHDF2-Hx
B
IL-11
pg/mL
MHCC97H-shCtrl
MHCC97H-shYTHDF2
Nx
Hx
C
Nx
Hx
shCtrl
shYTHDF2
YTHDF2
SerpinE2
p-STAT3
β-actin
D
shCtrl
shYTHDF2
IL-11
Serpin E2
IL-11 IOD
Serpin E2 IOD
E
Ythdf2F/F
Ythdf2LKO
IL-11
Serpin E2
IL-11 IOD
Serpin E2 IOD

Figure S6

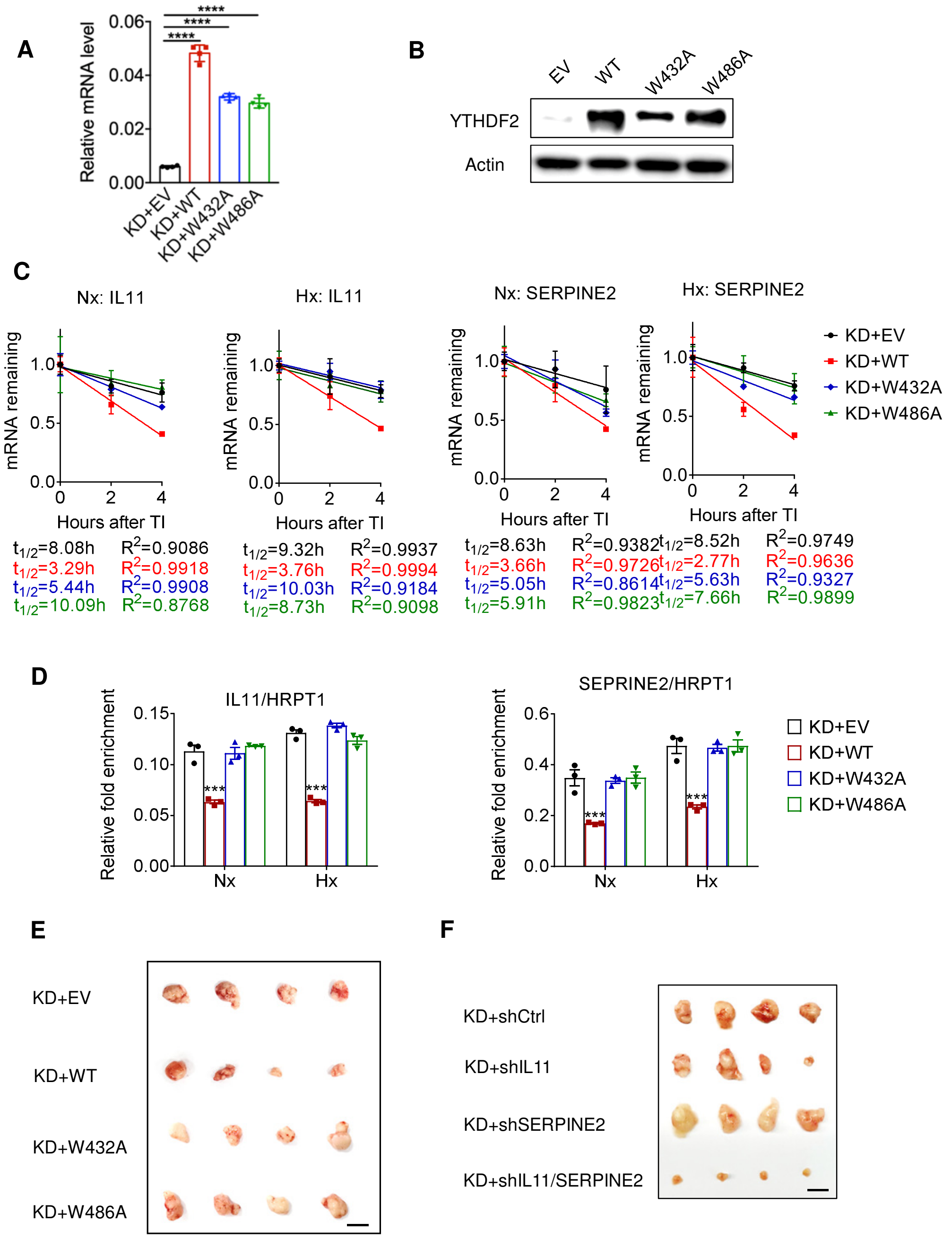

A
Relative mRNA level
0.06
0.04
0.02
0.00
****
****
****
KD+EV
KD+WT
KD+W432A
KD+W486A
B
EV
WT
W432A
W486A
YTHDF2
Actin
C
Nx: IL11
Hx: IL11
Nx: SERPINE2
Hx: SERPINE2
mRNA remaining
Hours after TI
KD+EV
KD+WT
KD+W432A
KD+W486A
t1/2=8.08h R2=0.9086
t1/2=3.29h R2=0.9918
t1/2=5.44h R2=0.9908
t1/2=10.09h R2=0.8768
t1/2=9.32h R2=0.9937
t1/2=3.76h R2=0.9994
t1/2=10.03h R2=0.9184
t1/2=8.73h R2=0.9098
t1/2=8.63h R2=0.9382
t1/2=3.66h R2=0.9726
t1/2=5.05h R2=0.8614
t1/2=5.91h R2=0.9823
t1/2=8.52h R2=0.9749
t1/2=2.77h R2=0.9636
t1/2=5.63h R2=0.9327
t1/2=7.66h R2=0.9899
D
IL11/HRPT1
SEPRINE2/HRPT1
Relative fold enrichment
***
Nx
Hx
KD+EV
KD+WT
KD+W432A
KD+W486A
E
KD+EV
KD+WT
KD+W432A
KD+W486A
F
KD+shCtrl
KD+shIL11
KD+shSERPINE2
KD+shIL11/SERPINE2

Figure S7

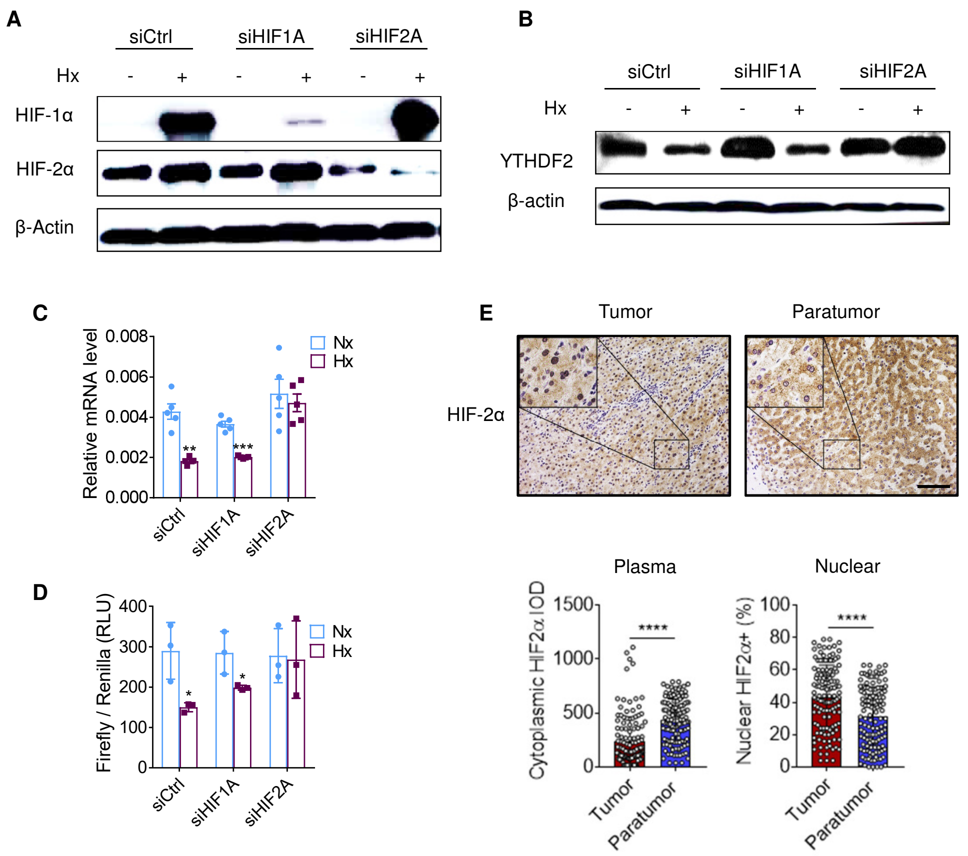
A
siCtrl
siHIF1A
siHIF2A
Hx
HIF-1α
HIF-2α
β-Actin
B
YTHDF2
β-actin
C
Relative mRNA level
Nx
Hx
D
Firefly / Renilla (RLU)
E
Tumor
Paratumor
HIF-2α
Plasma
Nuclear
Cytoplasmic HIF2α IOD
Nuclear HIF2α+ (%)

Figure S8

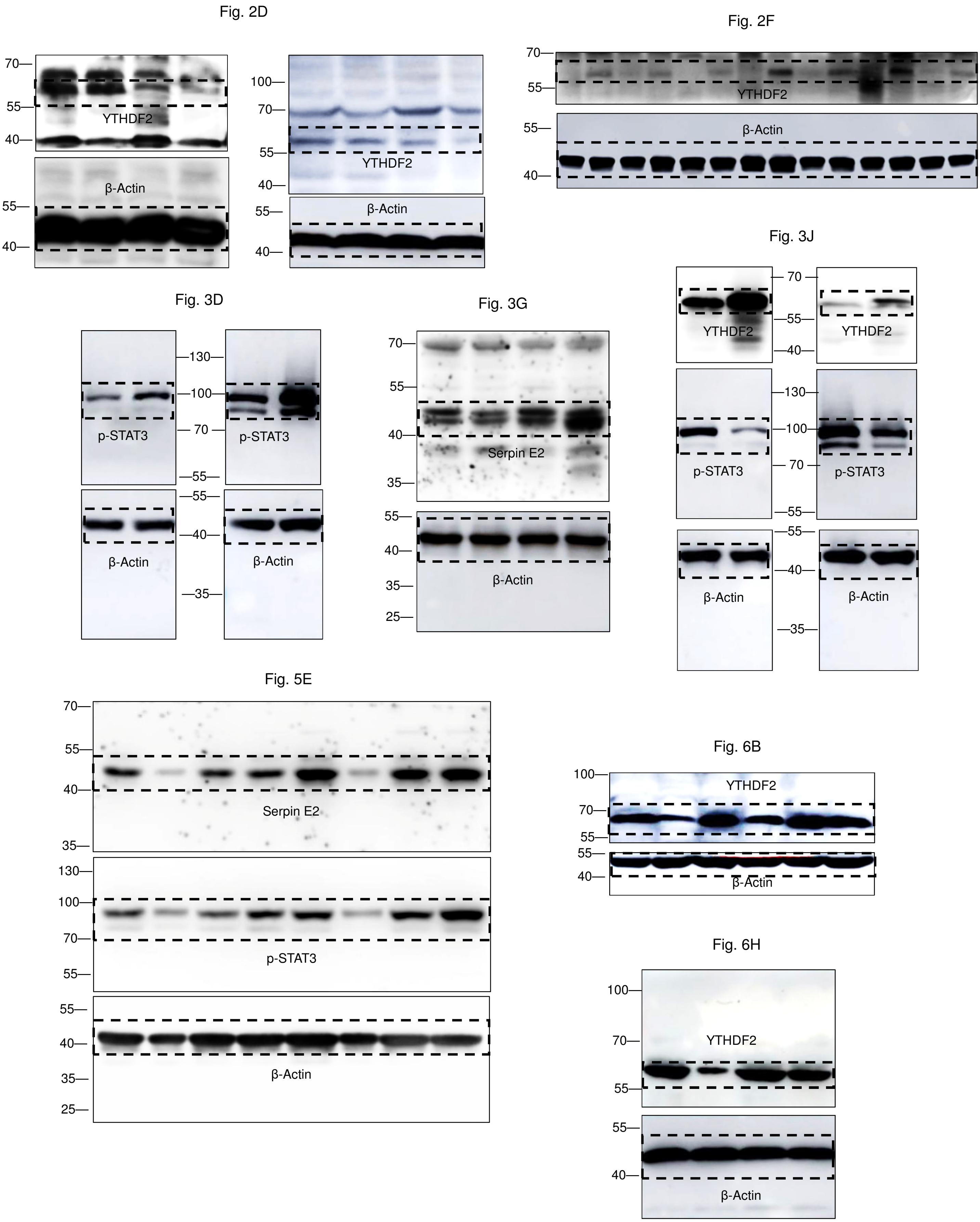

To be continued

Fig. 6J

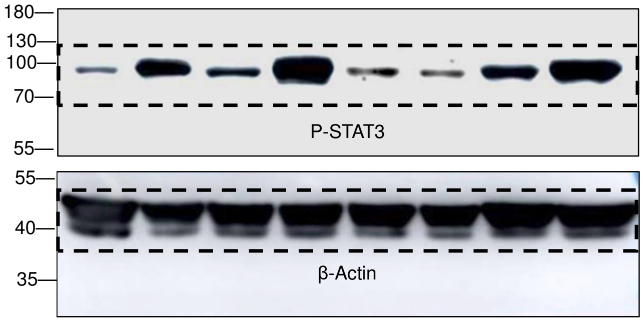

Supplementary Fig. 3B

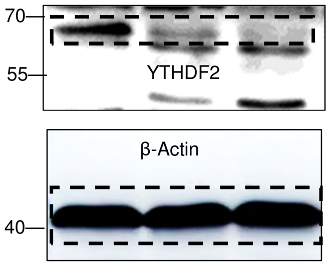

Supplementary Fig. 4B

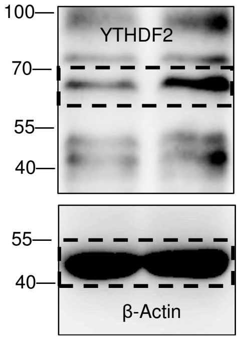

Supplementary Fig. 5C

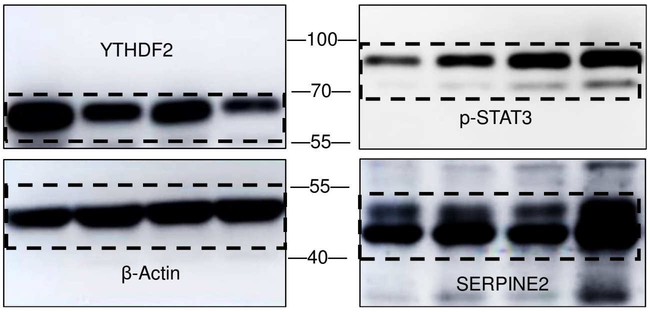

Supplementary Fig. 7A

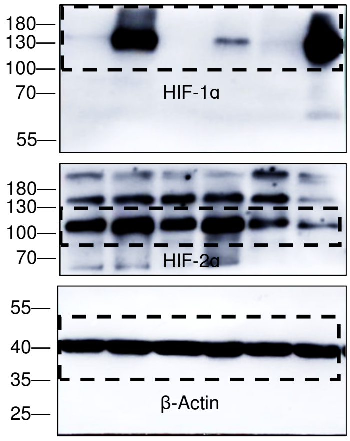

Supplementary Fig. 7B

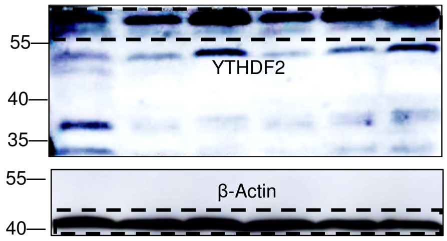

Supplement: Supplementary file 1 — Additional file 1: Supplementary Figures. Figure S1. Identification of a “hyper-up” pattern in m6A-epitranscriptome of human HCC. Figure S2. Expression of m6A modulators in human HCC tissues and hypoxic HCC cell lines. Figure S3. YTHDF2 deficiency enhances proliferative and proangiogenic functions of HCC cells. Figure S4. YTHDF2 inhibits tumor growth and vasculature remodeling. Figure S5. YTHDF2 deficiency upregulates IL-11 and Serpin E2 expression in HCC cells. Figure S6. YTHDF2 requires its recognitive function to degrade IL11 and SERPINE2 mRNAs. Figure S7. HIF-2α transcriptionally inhibits YTHDF2 expression in HCC cells. Figure S8. Unprocessed original scans of blots. [file 12943_2019_1082_MOESM1_ESM.pdf]
