# Supplementary material for: YTHDF2 reduction fuels inflammation and vascular abnormalization in hepatocellular carcinoma
Source: Mol Cancer. 2019 Nov 18;18:163. doi: 10.1186/s12943-019-1082-3 (PMC6859620; doi:10.1186/s12943-019-1082-3)
Supplement: Supplementary file 2 — Additional file 2: Table S1. Clinicopathological information of 200 HCC patients. [file 12943_2019_1082_MOESM2_ESM.docx]

**Table S1, Comparison of clinicopathological profiles of HCC patients**

| Variables |  | YTHDF2 level | | | | OR | | 95%CI | *P* Value |
| --- | --- | --- | --- | --- | --- | --- | --- | --- | --- |
|  |  | Low (n=100) | | High (n=100) | |  |  |  |  |
|  |  | No. of patients | % | No. of patients | % |  |  |  |  |
| **Age, yr** |  |  |  |  |  |  |  | |  |
| <51 |  | 49 | 49% | 47 | 47% | 1.803 | 0.622-1.887 | | 0.777 |
| ≥51 |  | 51 | 51% | 53 | 53% |  |  | |  |
| **Gender** |  |  |  |  |  |  |  | |  |
| Female |  | 21 | 21% | 14 | 14% | 1.633 | 0.778-3.429 | | 0.193 |
| Male |  | 79 | 79% | 86 | 86% |  |  | |  |
| **HBsAg** |  |  |  |  |  |  |  | |  |
| Negative |  | 16 | 16.5% | 6 | 6.1% | 3.062 | 1.144-8.194 | | **0.021** |
| Positive |  | 81 | 83.5% | 93 | 93.9% |  |  | |  |
| **Cirrhosis** |  |  |  |  |  |  |  | |  |
| Absent |  | 16 | 16.7% | 8 | 8.7% | 2.100 | 0.852-5.177 | | 0.102 |
| Present |  | 80 | 83.3% | 84 | 91.3% |  |  | |  |
| **Alpha-fetoprotein** |  |  |  |  |  |  |  | |  |
| ≤20ng/ml |  | 21 | 21.6% | 30 | 30.3% | 0.636 | 0.333-1.212 | | 0.167 |
| >20ng/ml |  | 76 | 78.4% | 69 | 69.7% |  |  | |  |
| **Tumor size** |  |  |  |  |  |  |  | |  |
| ≤5cm |  | 44 | 44% | 56 | 56% | 0.617 | 0.353-1.079 | | 0.090 |
| >5cm |  | 56 | 56% | 44 | 44% |  |  | |  |
| **Multinodular tumor** |  |  |  |  |  |  |  | |  |
| No |  | 64 | 66.7% | 75 | 90.6% | 0.453 | 0.231-0.891 | | **0.020** |
| Yes |  | 32 | 33.3% | 17 | 9.4% |  |  | |  |
| **Encapsulation** |  |  |  |  |  |  |  | |  |
| Complete |  | 39 | 40.6% | 43 | 46.7% | 1.283 | 0.720-2.285 | | 0.398 |
| None |  | 57 | 59.4% | 49 | 53.3% |  |  | |  |
| **Microvascular invasion** |  |  |  |  |  |  |  | |  |
| Negative |  | 54 | 56.3% | 65 | 70.7% | 0.534 | 0.292-0.976 | | **0.041** |
| Positive |  | 42 | 43.7% | 27 | 29.3% |  |  | |  |
| **Tumor grade** |  |  |  |  |  |  |  | |  |
| I-II |  | 36 | 37.5% | 38 | 41.3% | 0.853 | 0.475-1.531 | | 0.594 |
| III-IV |  | 60 | 62.5% | 54 | 58.7% |  |  | |  |
| **pTNM stage** |  |  |  |  |  |  |  | |  |
| Low (Stage I/II) |  | 42 | 51.9% | 52 | 77.6% | 0.331 | 0.151-0.639 | | **0.001** |
| High (Stage III) |  | 39 | 48.1% | 15 | 22.4% |  |  | |  |

*P* <0.05 was considered statistically significant, as was assessed by *χ*^2^-test. OR indicates odds ratio; 95% CI, 95% confidence interval; HBsAg, hepatitis B s-antigen; pTNM, pathologic tumor, lymph node, metastasis classification.
