# Supplementary material for: YTHDF2 reduction fuels inflammation and vascular abnormalization in hepatocellular carcinoma
Source: Mol Cancer. 2019 Nov 18;18:163. doi: 10.1186/s12943-019-1082-3 (PMC6859620; doi:10.1186/s12943-019-1082-3)
Supplement: Supplementary file 5 — Additional file 5: Supplementary Information. [file 12943_2019_1082_MOESM5_ESM.docx]

**Supplementary Information**

**Supplementary Materials and Methods**

**Cell culture**

HCC cell lines SMMC7721 and MHCC97H were obtained from Cell Resource Center of Shanghai Institutes for Biological Sciences, Chinese Academy of Sciences. The SMMC7721 cell line was validated through short tandem repeat (STR) profiling. LM3 and MHCC97L were kind gifts obtained from Huashan Hospital, Cancer Metastasis Institute, Fudan University. HUVEC and 293T cells were obtained from ATCC. All these cell lines were grown in DMEM medium (Gibco) supplemented with 10% fetal bovine serum (FBS) (Gibco), and 1% penicillin–streptomycin (Gibco), incubated at 37 °C and 5% CO_2_, and were tested for mycoplasma contamination once every three months. To render cultures hypoxic, HCC cell lines were incubated in an atmosphere of 1% O_2_, 5% CO_2_ and 94% N_2_. For *in vitro* HIF-2 antagonism, HCC cell lines were grown in the presence of 10 µM PT2385 (MedChem Express).

**Constructs and transfections**

Stable knockdown of YTHDF2, IL11 or SERPINE2 in HCC cell lines was generated by lentiviral-based shRNA delivery. Specific target shRNAs were subcloned into lentiviral vector pLKO.1 (Shanghai Genechem), while a non-target shRNA was used as a negative control. YTHDF2-overexpression construct was generated by subcloning PCR-amplified full-length cDNA into a GV358 (Ubi-MCS-Flag-SV40-EGFP-IRES-puromycin) lentiviral vector (Shanghai Genechem). An empty vector was used as a negative control. YTHDF2 mutants (W432A and W486A) were generated by site-directed mutagenesis. Viral particles were packaged in 293T cell and used to infect HCC cell lines in the presence of 8 µg/ml polybrene followed by puromycin selection. Knockdown or overexpression efficiency was confirmed at both mRNA and protein levels. The shRNA sequences employed in this study are listed as below: shYTHDF2#1, GAACGTCAAGGTCGTGGGAAA; shYTHDF2#2, ACACATTCGCCTAGAGAACAA; shIL11, GTGCACAGCTGAGGGACAAAT; shSERPINE2, GAACACAAAGAAACGCACTTT.

The siRNAs targeting HIF1A and HIF2A were transiently transfected into HCC cell lines by using Lipofectamine RNAiMAX (Invitrogen) following manufacturer’s instructions. A non-target siRNA was used as negative control. The siRNA sequences are listed as below: siHIF1A, GCUGAUUUGUGAACCCAUUTTAAUGGGUUCACAAAUCAGCTT; siHIF2A, GCGCAAAUGUACCCAAUGATTUCAUUGGGUACAUUUGCGCTT.

**Immunohistochemistry**

Immunohistochemistry for target molecules was performed on serial sections from tumor tissues of HCC patients and mice xenografts. Tissue sections were deparaffinized, subjected to antigen retrieval, and incubated with primary antibodies against YTHDF2 (Abcam, ab170118), IL11 (Proteintech, 55169-I-AP), Serpin E2 (Novus, NBP2-20315) and HIF-2ɑ (Abcam, ab199), CD31 (Abcam, ab28364) and Ki67 (Abcam, ab15580). All responses were followed by staining with the corresponding HRP-conjugated secondary antibody (Jackson Immuno Research Laboratories). The apoptotic tumor cells were defined by using In Situ Cell Death Detection Kit (Roche) following manufacturer’s protocols. The stained slides were assessed with integrated optical density (IOD) using ImageJ software. Tumor proliferation or apoptosis was quantified by calculating positively stained cells in at least five randomly chosen HPFs of each slide. Tumor microvessel areas were made of all distinct brown staining endothelial cells in the cancerous regions over five fields in each slide. The microvessel density (MVD) was defined using the average value of the five readings. For determination of vascular mimicry, the mouse tumor sections were stained with Periodic Acid-Schiff (PAS) Kit (Sigma-Aldrich) according to manufacturer’s instructions in addition to haematoxylin and eosin (H&E) staining and CD31 Immunohistochemistry. Vascular mimicry channels were assessed in five random HPFs per tumour with the criteria: (1) PAS^+^ channels that contain red cells and/or fluid, (2) the absence of CD31^+^ endothelial cells in the channels, and (3) tumour cells lining vascular spaces with no evidence of a matrix.

**Immunoblotting**

Cell or tissue lysates were separated on 6-8% polyacrylamide-SDS gels and transferred to a nitrocellulose membrane using transfer buffer (25 mM Tris, 192 mM glycine and 10% methanol). The blots were blocked with 5% non-fat milk in PBS containing 0.05% Tween-20 for 1 h and then probed overnight at 4°C in PBST with primary antibodies against YTHDF2 (Novus, NBP2-31785), p-STAT3 (CST, 9145S), Serpin E2 (Novus, NBP2-20315), HIF-1ɑ (Abcam, ab179483), HIF-2ɑ (Abcam, ab199). Next, the blots were incubated with a secondary antibody conjugated to horseradish peroxidase (HRP) (1:5,000, Jackson Immuno Research Laboratories) and detected with a ChemiDoc XRS system (Bio-Rad).

**Real-time quantitative PCR**

Total RNA samples used for RT-qPCR were isolated by using an RNeasy kit (ΒioTeke) with an additional on-column DNase-I digestion step. Total RNA or purified mRNA was reverse transcribed with PrimeScript™ RT Master Mix (Takara) using Oligo dT primers to obtain complementary DNA. qPCR was carried out by using SYBR Premix Ex Taq II (Takara). ACTIN (for mRNA expression level) and HPRT1 (for m^6^A-mRNA detection) were used as internal controls. The primers used in this study are: WTAP_F, GGCGAAGTGTCGAATGCT; WTAP_R, CCAACTGCTGGCGTGTCT; KIAA1429_F, AAGTGCCCCTGTTTTCGATAG; KIAA1429_R, ACCAGACCATCAGTATTCACCT; METTL3_F, AAGCTGCACTTCAGACGAAT; METTL3_R, GGAATCACCTCCGACACTC; METTL14_F, AGAAACTTGCAGGGCTTCCT; METTL14_R, TCTTCTTCATATGGCAAATTTTCTT; FTO_F, TGGGTTCATCCTACAACGG; FTO_R, CCTCTTCAGGGCCTTCAC; ALKBH5_F, CCCGAGGGCTTCGTCAACA; ALKBH5_R, CGACACCCGAATAGGCTTGA; YTHDF1_F, CTCCGATTCCATACCTCA; YTHDF1_R, AGCTGCTCCCATACGC; YTHDF2_F, TAGCCAACTGCGACACATTC; YTHDF2_R, CACGACCTTGACGTTCCTTT; YTHDF3_F, ATGCGTATGCTGGTGTC; YTHDF3_R, TTGAGGTGGTATGCTTGA; YTHDC1_F, TGGAGCCAGCCGTAAT; YTHDC1_R, GTATGTGACCCTCAGAAAGA; YTHDC2_F, GTGTCTGGACCCCATCCTTA; YTHDC2_R, CCCATCACTTCGTGCTTTTT; IL11_F, GAGCGGACCTACTGTCCTAC; IL11_R, CAGCCGAGTCTTCAGCAG; SERPINE2_F, TGGTGATGAGATACGGCGTAA; SERPINE2_R, GTTAGCCACTGTCACAATGTCTT ; ACTB_F, GGGAAATCGTGCGTGACATTAAG; ACTB_R, TGTGTTGGCGTACAGGTCTTTG; HPRT1_F, TGACACTGGCAAAACAATGCA; HPRT1_R, GGTCCTTTTCACCAGCAAGCT.

**Immunofluorescence**

Cells grown on glass slides were washed once in PBS and then fixed in 4% paraformaldehyde in PBST (PBS with 0.05% Tween-20) at room temperature for 15 min under rotation. The fixing solution was removed, and chilled methanol was immediately added to each chamber and incubated for 10 min at room temperature. The cells were rinsed once in PBS and incubated with blocking solution (10% FBS in PBST) for 1 h at room temperature under rotation. After that, the slides were incubated with primary antibodies against YTHDF2 (Abcam, ab170118), DCP1a (Sigma-Aldrich, WH0055802M6) at 4 °C overnight. Secondary antibodies (Alexa 488 and Alexa 647 conjugates) were incubated at room temperature for 1 h. After wash, mounting medium with DAPI (Santa Cruz) was added to mount the slides.

For fluorescent in situ hybridization (FISH) in HCC cells, RNA probes targeting human IL11 and SERPINE2 were conjugated with Cy3 and used according to the manufacturer’s instructions (Beijing Bilaige Biotechnology Co., Ltd). After the washing step, the sample preparation proceeded to the blocking step of the previous paragraph in the presence of RNase inhibitor.

Mouse tumors were fixed in formaldehyde and embedded in paraffin using standard procedures. Paraffin was removed and slides were rehydrated in two xylene baths (5 min), followed by five 3-min ethanol baths at decreasing concentrations (100%, 96%, 70%, 50% and 0%) and a 3-min TBS (50 mM Tris, 150 mM NaCl, pH 7.6) bath. The following antibodies were used: anti-YTHDF2 (Abcam, ab170118), anti-NG2 (Proteintech, 55027-I-AP), anti-CD31 (Abcam, ab28364).

To visualize vascular leakage in the primary tumor, 100 µl of dextran Alexa 647, 10 kDa (Life Technologies) was injected into NPG mice by tail vein injection. Three minutes later, mice were perfused with 4% paraformaldehyde (PFA). Tumors were collected and placed in 4% PFA overnight at 4 °C. After this, samples were infiltrated with 20% sucrose over-night at 4 °C. Tumors were frozen in OCT compound and 25-mm thick sections were cut, washed, then mounted with mounting medium with DAPI (Santa Cruz).

For *in vivo* hypoxia assessment, 2 mg pimonidazole (Hypoxyprobe) was injected intravenously into each tumor-bearing mouse and was left to circulate for 20 min before tumour resection. Staining of tumour sections was performed with a Hypoxyprobe Plus Kit according to manufacturer’s instructions.

Fluorescent images were captured using LSM780 Confocal microscope (Zeiss), and analyzed by ImageJ software.

**Cell proliferation, migration and mammosphere assay**

For cell proliferation assay, 1×10^3^ cells were seeded onto a 96-well plate. Cell number was monitored with Cell Counting Kit-8 (CCK-8) (Dojindo) for 5 consecutive days. To observe cell proliferation under hypoxia, 5000 cells were seeded per well in a 96-well plate. The cell proliferation was assessed after 72 h of hypoxia or normoxia culture by assaying the cells using the Cell Proliferation Reagent WST-1 (Sigma-Aldrich) following the manufacturer’s protocols. For each cell line tested, the signal was normalized to the value observed about 5 h after seeding.

For migration assay, 5×10^5^ cells were re-suspended in serum- free media and were seeded into the upper chamber of the transwell while FBS- containing media were added to the bottom chamber to provide chemoattractants for migration. After 24 h, migrated cells were fixed with methanol and stained with crystal violet for cell counting.

For mammosphere assay, cultured cells were trypsinized and then seeded in 24-well ultra-low attachment plates (Corning) at a density of 2,000 cells per milliliter in Complete MammoCult Medium (Stem Cell Technologies). After 2 weeks, the cells were photographed under microscope, and mammospheres (diameter ≥70 µm) were counted.

**Tube formation assay**

Each well of prechilled 96-well plates was coated with 50 μl/well of Matrigel (BD Biosciences) and incubated at 37 °C for 1 h. 1×10^4^ HUVEC and 2×10^4^ SMMC7721 in 100 μl medium were seeded onto the solidified gel. After incubated for 8 hrs, the endothelial tubes were counted under photomicroscope at HPF over five wells in each group.

**Enzyme-linked immunosorbent assay**

IL-11 concentration in cell culture medium was determined by using the human IL-11 enzyme-linked immunosorbent assay (ELISA) kit according to the manufacturer’s instructions (Raybiotech).

**Luciferase reporter assay**

The 3′ UTR fragments of IL11 (1261 ~ 1440 bp) and SERPINE2 (1501 ~ 1680 bp) containing putative m^6^A motifs were subcloned into luciferase reporter vector pmirGLO (Promega). SMMC7721 cells stably overexpressing YTHDF2 or EGFP were transfected with pmirGLO-IL11_3’UTR or pmirGLO-SERPINE2_3’UTR plasmid using Lipofectamine 2000 (Invitrogen). For promoter activity assay, a DNA fragment of *Ythdf2* (-1950 ~ -800 bp in the upstream of transcription start site) was subcloned into luciferase reporter vector pGL4 (Promega). SMMC7721 Cells were co-transfected with pGL4-*Ythdf2*_pro and siHIF1A, siHIF2A, or non-targeting siRNA. The pRL-SV40 *Renilla* luciferase reporter was transfected as an internal control. Firefly and *Renilla* luciferase activities were measured after 48 h using the dual-luciferase reporter assay system (Promega). *Renilla* luciferase activity was normalized to firefly activity and presented as relative luciferase activity.

**Chromatin immunoprecipitation**

Cells (70% confluence) from two 10-cm plates were washed with PBS then cross-linked with 1% formaldehyde for 10 min at room temperature. Fixation was stopped by 200mM glycine for 5 min. Cells were washed twice with PBS and harvested in lysis buffer (20mM Tris-HCl, pH 8.0, 150mM NaCl, 10mM EDTA, 1% SDS) containing proteinase inhibitors, 1mM DTT and 1mM PMSF. Lysates were sonicated on ice to yield 200-1000 bp genomic DNA fragments and then centrifuged at maximal speed, 4 °C for 10 min. And the samples were diluted 10 fold in dilution buffer and immunoprecipitated by anti-HIF2α antibody (2 μg) with 30 μl protein G beads over night at 4 °C. IgG as negative control was also used. The beads were collected and washed extensively. The immuno-complex was eluted with freshly prepared elution buffer (1% SDS, 100 mM NaCHO_3_) for 30 min at room temperature. Crosslinks were reversed with an incubation of the samples with 300 mM NaCl and proteinase K at 65 °C for 4 h. Immunoprecipitated DNA was purified using the PCR Purification Kit. Two microliters of the DNA samples were subjected into the q-PCR reactions. Primers used: -1898~-1698_F, CACCACGCTCAAGCTAATTT; -1898~-1698_R, ACCTACCCAACAGGTTTGTCGT; -1498~-1343_F, AAATCTGGGCCATGGAGCTAAG; -1498~-1343_R, ATCGGAGGCTGAGCAGGAGAAT; -1045~-872_F, TCCTTCACCTTTGGTGCCTG; -1045~-872_R, TCGTACTATCCACACGCTGG. The results were normalized for the signal of the input and were expressed as a percentage of the signal with the antibody.

**m^6^A-seq data analyses**

m^6^A-seq data were analyzed according to the protocol described by Meng *et al* [1]. Briefly, Tophat2 (version 2.2.1) with Bowtie1 [2, 3] support was run to align the sequence reads to reference genome and transcriptome (hg19). Then exomePeak R/Bioconductor package (version 3.7) [1] was used to find m^6^A peaks. Significant peaks with false discovery rate (FDR) less than 0.05 were annotated to RefSeq database (hg19). Sequence motifs were identified by using Homer (version 4.9) [4], and DAVID (version 6.8) was used to perform GO term enrichment analysis [5]. Gene expression was calculated by Cufflinks (version 2.2.1) using the sequencing reads from input samples [6]. Cuffdiff was used to find the differentially expressed genes [7].

**Supplementary Figure Legends**

**Figure S1.** **Identification of a “hyper-up” pattern in m^6^A-epitranscriptome of human HCC**

(A, B) m^6^A levels in paired tumor (T) versus paratumor (P) total RNA or mRNA, as assessed by dot blot (A) and quantified by grey intensity (B). *n* = 6 patients.

(C) Percentages of hypomethylated (hypo) and hypermethylated (hyper) m^6^A-mRNAs in tumor compared to paratumor, as determined by MeRIP-Seq. *n* = 8 patients.

(D, E) Gene numbers (D) and percentages (E) in different portions of m^6^A-mRNAs (classified as hypo-up, hypo-down, hyper-up and hyper-down) were individually assessed by MeRIP-Seq. *n* = 8 patients.

(F) GO enrichment map of high-frequency “hyper-up” genes (shared by at least 3 patients) as discovered in (C).

(G) GO analysis of hyper-upregulated genes in hypoxic SMMC7721 cells based on MeRIP-seq, showing the most significant GO terms and the *P* values.

(H) YTHDF2 mRNA levels in human HCC cell lines grown for 0, 6, 12 or 24 h under hypoxia, as assessed by RT-qPCR. *n* = 3 biological replicates.

(I) Immunoblot of YTHDF2 in human HCC cell line SMMC7721 (upper) and LM3 (lower) grown for 0, 6, 12 or 24 h under hypoxia. *n* = 2 independent experiments.

Error bars indicate means ± SEM **P* < 0.05, ***P* < 0.01, ****P* < 0.001. *P*-values were determined by two tailed *t*-test.

**Figure S2.** **Expression of m^6^A modulators in human HCC tissues and hypoxic HCC cell lines**

(A) mRNA levels of indicated m^6^A modulators in paired tumor and paratumor tissues were assessed using RT-qPCR. *n* = 48 patients.

(B) mRNA levels of indicated m^6^A modulators in SMMC7721 cells grown for 0, 6, 12 or 24 h under hypoxia, as assessed by qPCR. *n* = 3 biological replicates.

(C) Immunohistological staining of YTHDF2 in tumor core, margin and paratumor tissues, as quantified by integrated optical density (IOD) value. *n* = 101-103 patients. Scale bar, 100 µm.

(D) Percentages of Barcelona Clinic Liver Cancer (BCLC) classified patients in YTHDF2^Low^ and YTHDF2^High^ categories, as determined through immunohistological staining. Scale bar, 100 µm.

Error bars indicate means ± SEM **P* < 0.05, ***P* < 0.01, ****P* < 0.001, *****P* < 0.0001. *P*-values were determined by two tailed *t*-test.

**Figure S3.** **YTHDF2 deficiency enhances proliferative and proangiogenic functions of HCC cells**

(A, B) SMMC7721 cells were transduced with a control shRNA (shCtrl) or shRNAs targeting YTHDF2 (shYTHDF2#1&#2). YTHDF2 knockdown was confirmed in mRNA and protein levels, as measured using RT-qPCR (A) and western blot (B). *n* = 4 biological replicates (A) or 2 independent experiments (B).

(C) Trypan blue staining of dead SMMC7721-shCtrl or SMMC7721-shYTHDF2 cells grown under normoxia (Nx) or hypoxia (Hx). *n* = 3 biological replicates.

(D) Proliferative activity of MHCC97H-shCtrl and MHCC97H-shYTHDF2 cells grown for 4 d as assessed by CCK8 assay. *n* = 4-5 biological replicates.

(E) Numbers of endothelial tubes formed by HUVEC cocultured with indicated MHCC97H cells under Nx or Hx for 24 h. *n* = 3 biological replicates.

(F) Migration ability of indicated SMMC7721 cells under Nx or Hx as determined by transwell assay. *n* = 3 biological replicates.

(G) Stemness of indicated SMMC7721 cells as determined by sphere formation assay. *n* = 4 biological replicates.

(H) Metabolism of indicated SMMC7721 cells as determined by lactate production assay. *n* = 3 biological replicates.

(I, K) Proliferative ability of subcutaneous tumors derived from indicated SMMC7721 (I) or MHCC97H (K) cells, as assessed using immunohistological staining of Ki67. *n* = 5 mice. Scale bar, 100 µm.

(J, L) Vessel permeability of subcutaneous tumors derived from indicated SMMC7721 (J) or MHCC97H (L) cells, as assessed by dextran staining. *n* = 5 mice. Scale bar, 100 µm.

Error bars indicate means ± SEM **P* < 0.05, ***P* < 0.01, ****P* < 0.001, **** *P* <0.0001. *P*-values were determined by two tailed *t*-test.

**Figure S4.** **YTHDF2 inhibits tumor growth and vasculature remodeling**

(A, B) SMMC7721 cells were transduced with an empty vector (EV) or vector overexpressing YTHDF2 (OE). YTHDF2 knockdown or overexpression was confirmed in mRNA and protein levels, as measured using RT-qPCR (A) and western blot (B). *n* = 4 biological replicates (A) or 2 independent experiments (B).

(C) Proliferative activity of SMMC7721-NC and SMMC7721-OE cells grown for 4 d as assessed by CCK8 assay. *n* = 6 biological replicates.

(D) Numbers of endothelial tubes formed by HUVEC cocultured with indicated SMMC7721 cells under Nx or Hx for 24 h. *n* = 3 biological replicates.

(E) SMMC7721-NC and SMMC7721-OE cells were injected subcutaneously into NPG mice and tumor volumes were measured at indicated dates. *n* = 5 mice.

(F) Proliferative ability of subcutaneous tumors derived from indicated SMMC7721 cells, as assessed using immunohistological staining of Ki67. *n* = 5 mice. Scale bar, 100 µm.

(G) Microvessel density (MVD) in SMMC7721-derived tumors, as assessed by CD31 staining and quantification of microvessel areas. *n* = 3 mice. Scale bar, 100 µm.

(H) Vessel permeability of subcutaneous tumors derived from indicated SMMC7721 cells, as assessed by dextran staining. *n* = 5 mice. Scale bar, 100 µm.

(I-K) Tumor bearing livers were harvested from 8.5-month old *Ythdf2^F/F^* and *Ythdf2^LKO^* mice injected with DEN and CCL4. Tissue sections were stained for H&E (I), Ki67 (J), TUNEL (K). *n* = 4 mice. Scale bar, (I) 200 µm, (J, K) 100 µm.

Error bars indicate means ± SEM **P* < 0.05, ***P* < 0.01, ****P* < 0.001, **** *P* <0.0001. *P*-values were determined by two tailed *t*-test.

**Figure S5.** **YTHDF2 deficiency upregulates IL-11 and Serpin E2 expression in HCC cells**

(A) RT-qPCR analysis of the relative mRNA levels of selected genes in indicated SMMC7721 (upper) or MHCC97H (lower) cells grown for 24 h under Nx or Hx. *n* = 3 biological replicates.

(B) ELISA analysis of the quantitative protein levels of IL-11 in the culture supernatant of indicated MHCC97H cells. *n* = 3 biological replicates.

(C) Immunoblots showing STAT3 phosphorylation and Serpin E2 expression in indicated MHCC97H cells grown for 24 h under Nx or Hx. *n* = 2 independent experiments.

(D) Immunohistological staining of IL-11 and Serpin E2 in indicated SMMC7721-derived mouse tumors. *n* = 5 mice. Scale bar, 100 µm.

(E) Tumor bearing livers were harvested from 8.5-month old *Ythdf2^F/F^* and *Ythdf2^LKO^* mice injected with DEN and CCL4. Tissue sections were stained for IL-11 (upper) and Serpin E2 (lower) using immunohistochemistry. *n* = 4 mice. Scale bar, 50 µm.

Error bars indicate means ± SEM **P* < 0.05, ***P* < 0.01, ****P* < 0.001, **** *P* <0.0001. *P*-values were determined by two tailed *t*-test.

**Figure S6. YTHDF2 requires its recognitive function to degrade IL11 and SERPINE2 mRNAs**

(A, B) An empty vector (EV) or vectors encoding wild-type (WT) or mutant YTHDF2 (W432A and W486A) were transduced into SMMC7721 cells with a YTHDF2-knockdown (KD) background. YTHDF2 overexpression was confirmed in mRNA and protein levels, as measured using RT-qPCR (A) and western blot (B). *n* = 4 biological replicates (A) or 2 independent experiments (B).

(C) RNA lifetime of IL11 and SERPINE2 in indicated SMMC7721 cells, as determined by monitoring transcript abundance after transcription inhibition (TI). The cells were subjected to Nx or Hx 8 h prior to TI initiation. *n* = 3 biological replicates.

(D) m^6^A enrichment in the IL11 and SERPINE2 mRNAs in indicated SMMC7721 cells, as assessed by MeRIP-qPCR. *n* = 3 biological replicates.

(E, F) Representative photos of subcutaneous tumors derived from the above cell lines (E) or cell lines transduced with indicated shRNAs (F). Scale bar, 1 cm.

Error bars indicate means ± SEM ****P* < 0.001. *P*-values were determined by two tailed *t*-test.

**Figure S7. HIF-2α transcriptionally inhibits YTHDF2 expression in HCC cells**

(A) SMMC7721 cells were transduced with a control siRNA (siCtrl) or siRNA targeting HIF-1/2α. HIF-1/2α knockdown was confirmed using western blot. *n* = 2 biological replicates.

(B) Immunoblot of YTHDF2 in indicated LM3 cells expressing indicated siRNAs after 24 h of Nx or Hx exposure. *n* = 2 independent experiments.

(C) RT-qPCR analysis of YTHDF2 in LM3 cells expressing indicated siRNAs after 24 h of Nx or Hx exposure.

(D) *Ythdf2* promoter activity in LM3 cells expressing indicated siRNAs, as quantified using luciferase assay. *Renilla* luciferase activity was normalized to firefly activity and presented as relative luciferase activity. *n* = 3 biological replicates.

(E) Immunohistological staining of HIF-2α in human HCC tumor and paratumor tissues (upper), as quantified by integrated optical density (IOD) value (Plasma) or positive cell ratios (Nuclear) (lower). *n* = 130 patients. Scale bar, 50 µm.

Error bars indicate means ± SEM **P* < 0.05, ***P* < 0.01, ****P* < 0.001, *****P* < 0.0001. *P*-values were determined by two tailed *t*-test.

**Figure S8. Unprocessed original scans of blots.** Unprocessed images of all immunoblots. Molecular weight markers in kDa.

**Supplementary Table Legends**

**Table S1.** **Clinicopathological information of 200 HCC patients**

**Table S2.** **Upregulated genes in YTHDF2-deficient SMMC7721 cells**

RNA profiles of SMMC7721-shYTHDF2 and SMMC7721-shCtrl cells were assessed by RNA-seq. *P* value of 0.05 is used as the cutoff.

**Table S3.** **Downregulated genes in YTHDF2-overexpressed SMMC7721 cells**

SMMC7721-OE versus SMMC7721-EV cells were grown for 12 h under hypoxia and RNA profiles were assessed by RNA-seq. *P* value of 0.05 is used as the cutoff.

**Supplementary References**

1. Meng J, Lu Z, Liu H, Zhang L, Zhang S, Chen Y, Rao MK, Huang Y: **A protocol for RNA methylation differential analysis with MeRIP-Seq data and exomePeak R/Bioconductor package.** *Methods* 2014, **69:**274-281.

2. Kim D, Pertea G, Trapnell C, Pimentel H, Kelley R, Salzberg SL: **TopHat2: accurate alignment of transcriptomes in the presence of insertions, deletions and gene fusions.** *Genome Biol* 2013, **14:**R36.

3. Langmead B, Trapnell C, Pop M, Salzberg SL: **Ultrafast and memory-efficient alignment of short DNA sequences to the human genome.** *Genome Biol* 2009, **10:**R25.

4. Heinz S, Benner C, Spann N, Bertolino E, Lin YC, Laslo P, Cheng JX, Murre C, Singh H, Glass CK: **Simple combinations of lineage-determining transcription factors prime cis-regulatory elements required for macrophage and B cell identities.** *Mol Cell* 2010, **38:**576-589.

5. Huang da W, Sherman BT, Lempicki RA: **Systematic and integrative analysis of large gene lists using DAVID bioinformatics resources.** *Nat Protoc* 2009, **4:**44-57.

6. Trapnell C, Williams BA, Pertea G, Mortazavi A, Kwan G, van Baren MJ, Salzberg SL, Wold BJ, Pachter L: **Transcript assembly and quantification by RNA-Seq reveals unannotated transcripts and isoform switching during cell differentiation.** *Nat Biotechnol* 2010, **28:**511-515.

7. Trapnell C, Hendrickson DG, Sauvageau M, Goff L, Rinn JL, Pachter L: **Differential analysis of gene regulation at transcript resolution with RNA-seq.** *Nat Biotechnol* 2013, **31:**46-53.
